# Genetic variants that modify neuroendocrine gene expression and foraging behavior of *C. elegans*

**DOI:** 10.1126/sciadv.adk9481

**Published:** 2024-06-12

**Authors:** Harksun Lee, Sonia A. Boor, Zoë A. Hilbert, Joshua D. Meisel, Jaeseok Park, Ye Wang, Ryan McKeown, Sylvia E. J. Fischer, Erik C. Andersen, Dennis H. Kim

**Affiliations:** ^1^ Division of Infectious Diseases, Department of Pediatrics, Boston Children’s Hospital and Harvard Medical School, Boston, MA 02115, USA.; ^2^ Department of Biology, Massachusetts Institute of Technology, Cambridge, MA 02139, USA.; ^3^ Department of Molecular Biosciences, Northwestern University, Evanston, IL 60208, USA.; ^4^ Harvard Medical School Initiative for RNA Medicine, Boston, MA 02115, USA.; ^5^ Department of Biology, Johns Hopkins University, Baltimore, MD 21212, USA.

## Abstract

The molecular mechanisms underlying diversity in animal behavior are not well understood. A major experimental challenge is determining the contribution of genetic variants that affect neuronal gene expression to differences in behavioral traits. In *Caenorhabditis elegans*, the neuroendocrine transforming growth factor–β ligand, DAF-7, regulates diverse behavioral responses to bacterial food and pathogens. The dynamic neuron-specific expression of *daf-7* is modulated by environmental and endogenous bacteria-derived cues. Here, we investigated natural variation in the expression of *daf-7* from the ASJ pair of chemosensory neurons. We identified common genetic variants in *gap-2*, encoding a Ras guanosine triphosphatase (GTPase)–activating protein homologous to mammalian synaptic Ras GTPase-activating protein, which modify *daf-7* expression cell nonautonomously and promote exploratory foraging behavior in a partially DAF-7–dependent manner. Our data connect natural variation in neuron-specific gene expression to differences in behavior and suggest that genetic variation in neuroendocrine signaling pathways mediating host-microbe interactions may give rise to diversity in animal behavior.

## Teaser

Molecular basis of natural variation in foraging behavior is revealed by genetic variants that modulate neuronal gene expression.

## INTRODUCTION

The molecular characterization of natural variants that affect behavioral traits provides a starting point to understand the cellular and organismal mechanisms driving diversity in behavior (*1*–*3*). How variation in gene expression might be manifest in differences in behavior has been relatively unexplored in part because the relationship between neuronal gene expression and behavioral states is not well understood. Activity-dependent neuronal transcription has been shown to have pivotal roles in the development and plasticity of neural circuits, but less is known about how changes in gene expression play a role in shaping behavioral states (*4*). Moreover, whereas variation in levels of gene expression have been readily quantified on a genome-wide scale across evolutionarily diverse organisms in many tissues, including the nervous system, mechanistically bridging changes in levels of gene expression to differences in phenotypic traits has continued to be a major challenge (*5*–*8*).

In *Caenorhabditis elegans*, a neuroendocrine transforming growth factor–β ligand, DAF-7, controls the dauer developmental decision (*9*, *10*) and diverse aspects of physiology including feeding and foraging behaviors (*10*–*16*). Neuron-specific expression of *daf-7* is modulated by endogenous and environmental bacterial cues (*9*, *10*, *16*–*21*), and we have observed that expression of *daf-7* from the ASJ pair of chemosensory neurons is differentially regulated in a range of physiological contexts (*16*, *18*, *21*). We showed that secondary metabolites produced by pathogenic *Pseudomonas aeruginosa* induced *daf-7* expression in the ASJ neurons, promoting avoidance behavior and increased roaming activity (*16*, *18*). In addition, we observed that male *C. elegans* were observed to up-regulate expression of *daf-7* from the ASJ neurons upon the onset of reproductive maturity, promoting bacterial food-leaving mate-searching behavior (*21*). Most recently, we observed that expression of *daf-7* from the ASJ neurons of adult hermaphrodite animals was regulated by interoceptive bacteria-derived cues from ingested food, defining a feedback loop mechanism that governs foraging behavior in response to the ingestion of bacterial food (*16*). Our studies have established that *daf-7* neuroendocrine gene expression modulates foraging behavior of *C. elegans* in response to environmental and internal cues from pathogenic and nutritional bacteria, and moreover, the expression of *daf-7* from the ASJ neurons is correlated with internal state favoring exploratory behavior (*16*). Here, we investigated genetic variation in *daf-7* expression from the ASJ neurons among wild strains of *C. elegans*, connecting differences in neuronal gene expression to diversity in foraging behavior that is manifest in natural populations.

## RESULTS

**Fig. 1.**
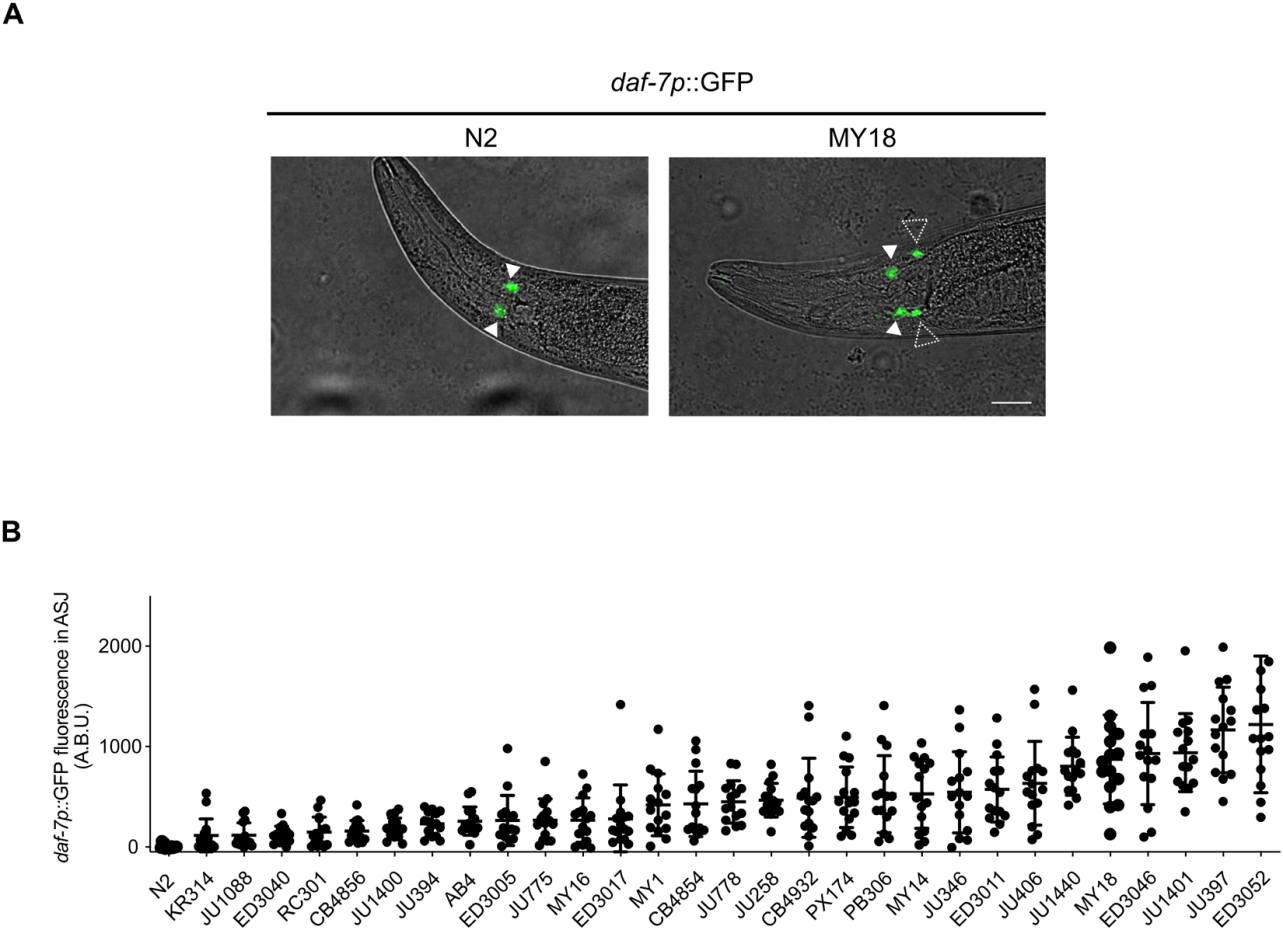
Natural variation in *daf-7* gene expression from the ASJ neurons in wild strains of *C. elegans*. (**A**) *daf-7p*::GFP expression pattern in N2 (left) and MY18 (right) genetic backgrounds. Filled triangles indicate the ASI neurons; dashed triangles indicate the ASJ neurons. Scale bar, 20 μm. (**B**) Maximum fluorescence values of *daf-7p*::GFP in the ASJ neurons in the genetic background of indicated wild strains. Each dot represents an individual animal, and error bars indicate SDs. Statistical test result with unpaired *t* test with N2 is in table S1.

We surveyed a set of wild strains of *C. elegans* for relative levels of *daf-7* expression in the ASJ neurons while feeding on the standard laboratory bacterial food *Escherichia coli* OP50. Previously, we found that for the laboratory wild-type strain N2 from Bristol, England, hermaphrodites do not exhibit detectable *daf-7* expression in ASJ neurons when feeding on *E. coli* OP50, but we have observed dynamic *daf-7* ASJ expression under different conditions, including the observation of *daf-7* ASJ expression in N2 hermaphrodites exposed to *P. aeruginosa* PA14 (*18*) and in N2 males feeding on *E. coli* OP50 (*21*). A genome-wide analysis of whole-animal gene expression levels across wild strains showed variation in *daf-7* expression (*8*). We observed a wide range of expression levels of *daf-7* in the ASJ neurons among wild strains sampled, with N2 exhibiting the lowest (undetectable) level of *daf-7* ASJ expression (Fig. 1, A and B). Our survey was conducted using strains generated from a cross of an integrated transgenic multicopy fluorescent reporter under the control of the *daf-7* promoter (derived from the N2 strain) into each wild-strain background. Our experimental approach thus precluded the observation of variation in *daf-7* expression due to local cis-acting loci because the reporter only had the regulatory regions of the N2 *daf-7* gene.

### Identification of natural variants in *gap-2* modulating *daf-7* expression in the ASJ neurons

**Fig. 2.**
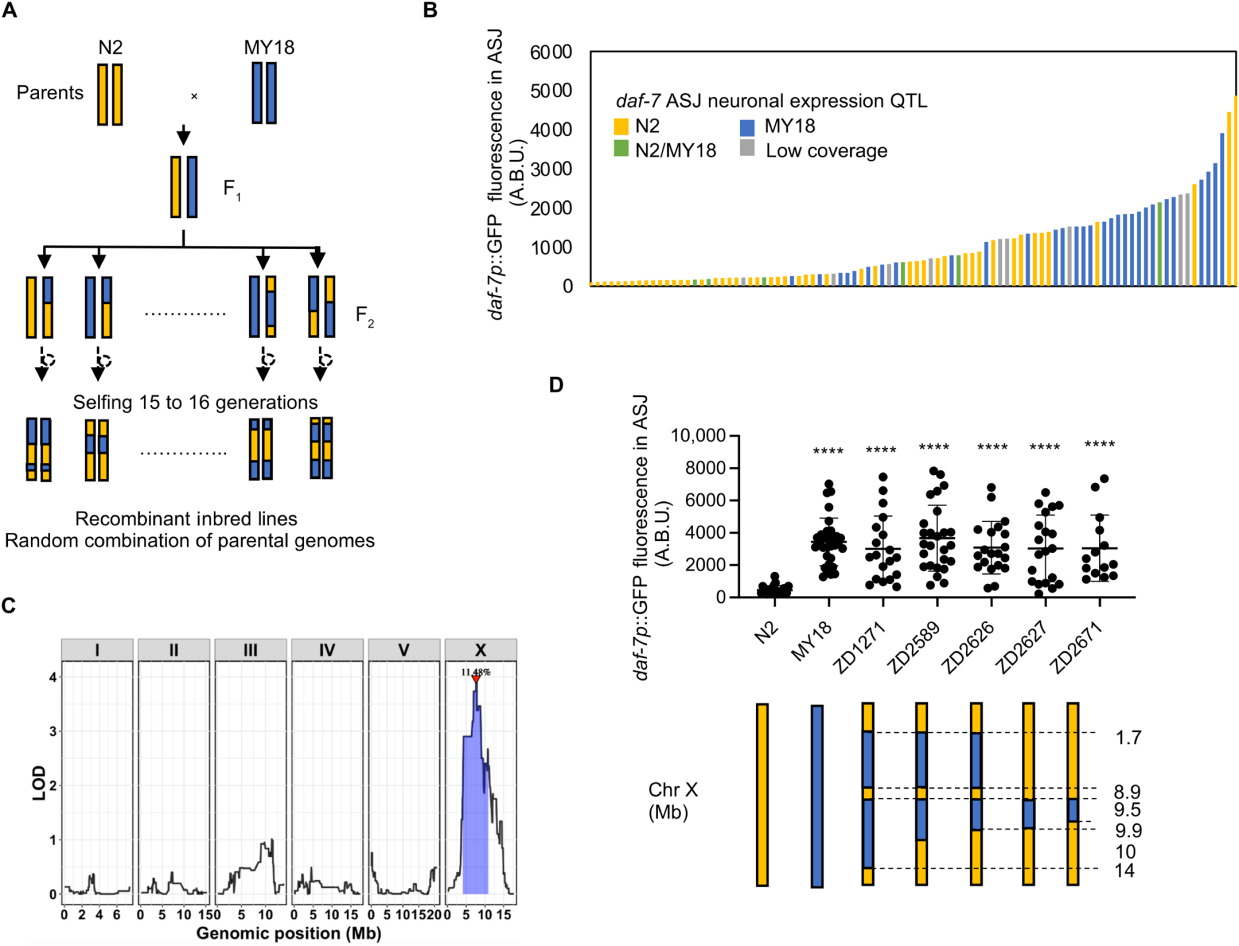
Identification of a genomic locus on chromosome X that affects *daf-7* expression in the ASJ neurons. (**A**) Diagram for constructing RILs with strains derived from N2 and MY18 backgrounds. (**B**) Maximum fluorescence values *daf-7p*::GFP in the ASJ neurons of 123 RILs and parental strains derived from N2 and MY18. The color of the bar represents the genotype at the QTL on chromosome X. (**C**) Linkage mapping results for *daf-7p*::GFP expression in the ASJ neurons. *X*-axis tick marks denote every 5 Mb. Significant QTL is denoted by a red triangle at the peak marker, and blue shading shows 95% confidence interval around the peak marker. The 5% genome-wide error rate logarithm of the odds (LOD) threshold is represented as a dashed horizontal line. (**D**) Maximum fluorescence values *daf-7p*::GFP in the ASJ neurons of NILs. Each dot represents an individual animal, and error bars indicate SDs. ****P < 0.0001 as determined by an unpaired two-tailed *t* test compared to N2.

To define a molecular basis of natural variation in *daf-7* ASJ expression, we focused on differences in *daf-7* ASJ expression between N2 and MY18, a strain isolated from Münster, Germany. We generated 123 recombinant inbred lines (RILs) from multiple crosses between the N2- and MY18-derived strains (Fig. 2A) and quantified levels of *daf-7* ASJ expression in each RIL strain (Fig. 2B). RIL sequencing of MY18 was used to identify 5716 variants that differed between the two parent strains (N2 and MY18), and a linkage mapping pointed to a broad quantitative trait locus (QTL) on chromosome X that contributed 11.5% of the observed differences in *daf-7* ASJ expression (Fig. 2C). In parallel, starting with an MY18-derived strain, we carried out multiple backcrosses with N2, selecting at each generation for the expression of *daf-7* ASJ expression, yielding near-isogenic lines (NILs) in the N2 genetic background defining a 447-kb interval on chromosome X: 9,513,008 to X: 9,960,248 containing an MY18-derived locus that conferred increased *daf-7* ASJ expression in adult hermaphrodites feeding on *E. coli* OP50 (Fig. 2D).

**Fig. 3.**
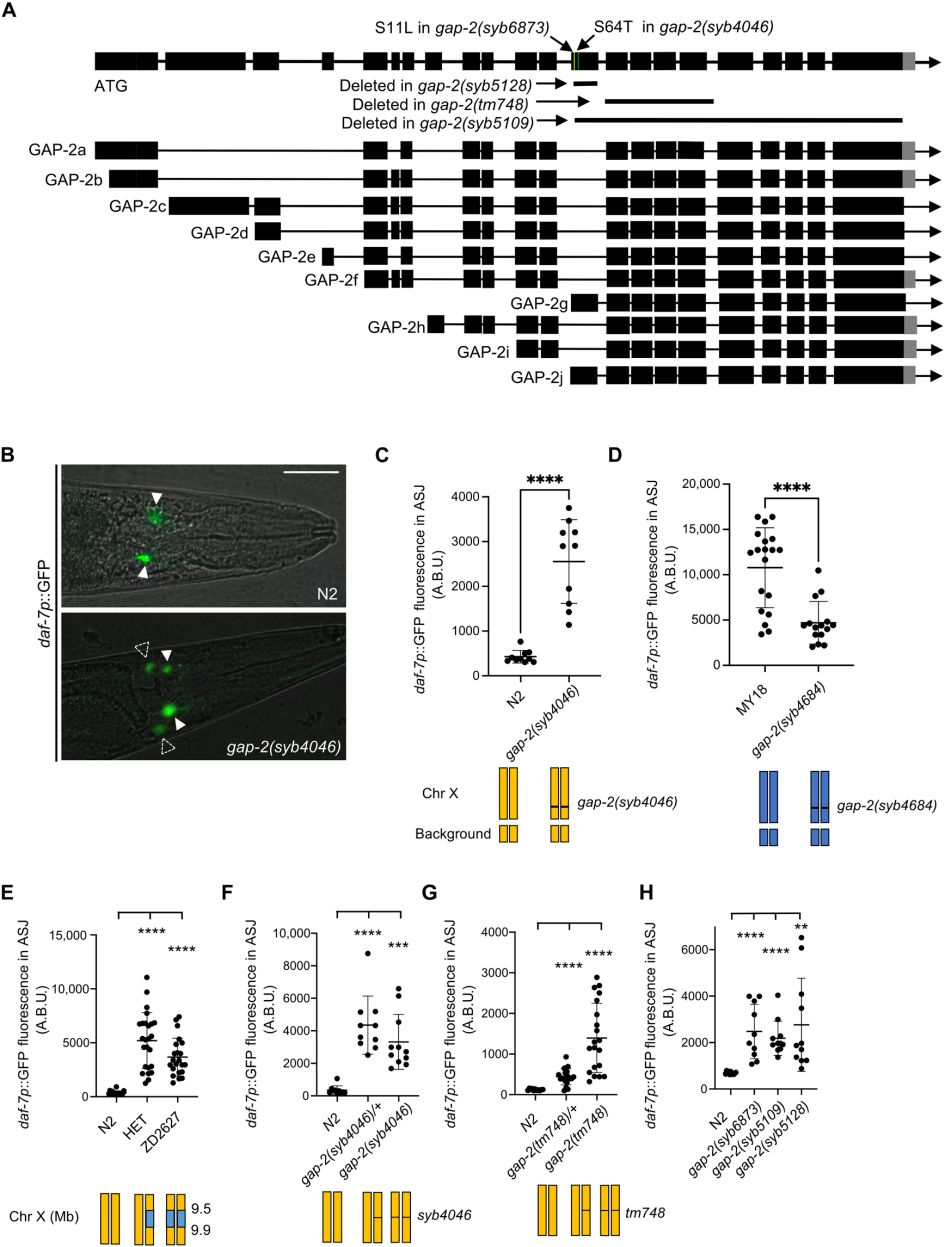
Natural variants in *gap-2* modulate *daf-7* expression in the ASJ neurons. (**A**) Genomic organization of multiple splice isoforms of *gap-2*. Deletion alleles of *gap-2* and the S11L change in *gap-2(syb6873)* and S64T change in *gap-2(syb4046)* are depicted. (**B**) Representative images of *daf-7p*::GFP expression in N2 and *gap-2(syb4046)*. Filled triangles indicate the ASI neurons; dashed triangles indicate the ASJ neurons. Scale bar, 20 μm. (**C** to **H**) Maximum fluorescence values *daf-7p*::GFP in the ASJ neurons of indicated strains. HET is heterozygous of N2 and NIL, ZD2627. Each dot represents an individual animal, and error bars indicate SDs. ****P < 0.0001 and **P < 0.01 as determined by an unpaired two-tailed *t* test. Each genotype was compared to N2.

To identify the specific nucleotide differences contributing to the differential *daf-7* ASJ expression between the N2 and MY18 strains, we adopted a candidate variant approach, examining nucleotide differences predicted to cause coding changes in the 18 genes present in the narrowed interval on chromosome X. One of these genes was *gap-2*, encoding a conserved Ras GTPase-activating protein (RasGAP) homologous to the synaptic Ras GTPase-activating protein (SynGAP) family. We identified a candidate variant present in MY18 that was predicted to affect an exon specific to two of the alternatively spliced isoforms of *gap-2*, *gap-2g*, and *gap-2j* and cause a putative substitution of a threonine in place of the serine at position 64 of GAP-2G and GAP-2J. First, we observed that a strain carrying the *gap-2(tm748)* deletion allele in the N2 background exhibited up-regulated *daf-7* ASJ expression in adult hermaphrodite animals feeding on *E. coli* OP50 (Fig. 3G), leading us to evaluate the effect of the 64T variant in the N2 background. We observed that a strain carrying the *gap-2(syb4046)* allele, which had been engineered to carry the S64T change in the *g*-/*j*- isoforms of *gap-2* in the N2 genetic background, exhibited increased *daf-7* ASJ expression on *E. coli* OP50 (Fig. 3, B and C). Conversely, when we examined the effect of engineering the T64S reciprocal change into the MY18 genetic background, we observed that *daf-7* ASJ expression decreased compared to what was observed in the MY18 strain (Fig. 3D). These data established that the S64T change in GAP-2 G-/J- isoforms caused increased *daf-7* ASJ expression. We observed that the *gap-2(syb4046)* allele causes a dominant phenotype (Fig. 3F), where the *gap-2(tm748)* heterozygote conferred an intermediate level of *daf-7* ASJ expression compared to the wild-type or *gap-2(tm748)* homozygous strains (Fig. 3G).

We observed that 48 other wild strains carry the 64T variant (Table 1) (*22*). On the basis of the putative alteration of specifically *gap-2g* and *gap-2j* isoforms by the 64T variants, we examined whether other variants present in the affected exon specific to *gap-2g* and *gap-2j* isoforms might also influence *daf-7* ASJ expression. We found that 24 wild strains harbor a variant that causes a putative S11L change (Table 1). To test the functional consequences of the S11L substitution in GAP-2, we generated the *gap-2(syb6873)* allele, in which the 11L variant was engineered in the N2 genetic background and observed that the 11L variant also conferred increased *daf-7* ASJ expression to a degree comparable to what was conferred by the 64T variant (Fig. 3H).

**Table 1.** Wild strains of *C. elegans* with common variants of *gap-2*. Database search from cendR variant annotation (*22*).

Strains with 64T GAP-2 G/J*	**Strains with 11L GAP-2 G/J^†^**
DL200, JU1213, JU1242, JU1400, JU1530, JU1666, JU1808, JU2001, JU2106, JU2131, JU2250, JU2478, JU2534, JU2566, JU2570, JU2575, JU2576, JU310, JU311, JU3128, JU3132, JU3140, JU3144, JU323, JU3795, JU440, JU792, JU847, MY16, MY18, MY2147, MY2453, MY2573, MY2741, MY772, MY795, NIC1, NIC1049, NIC1107, NIC166, NIC1698, NIC277, NIC3, NIC501, WN2064, WN2066, WN2086, WN2117	ECA369, JU258, JU2838, JU3166, JU3167, JU3169, MY1, NIC2021, NIC2076, NIC252, NIC266, NIC267, NIC268, NIC269, NIC274, NIC276, QG2813, QG2832, QG2837, QG2841, QG2846, QG4228, WN2063, XZ1735

*Strains carrying the 64T GAP-2 G/J variant have A instead of T in the locus of Chromosome X:9513,008.

†Strains carrying the 11L GAP-2 G/J variant have T instead of C in the locus of Chromosome X:9,512,850.

We observed that the effect of the *gap-2* variants on *daf-7* ASJ expression was large relative to a small increase in *daf-7* ASJ expression conferred by the *npr-1* (215F) change (fig. S1). We also noted that the *gap-2* variants did not cause a change in *daf-7* expression from the ASI pair of chemosensory neurons (fig. S2).

### *gap-2* natural variants causing differences in a foraging behavioral trait

Having established that two *gap-2* variants promoted *daf-7* ASJ expression, we sought to connect these variants that alter gene expression to corresponding *daf-7*–dependent behavioral traits. *C. elegans* exhibits a two-state foraging behavior that alternates between an exploratory roaming state and an exploitative dwelling state on bacterial food (*11*, *23*). A QTL affecting *exp-1* (*24*) and a laboratory-derived mutation in *npr-1* (*25*) have been previously shown to modify the proportion of time that animals spend in the roaming and dwelling states. *daf-7* mutants have been shown to spend an increased proportion of time in the dwelling state compared to the N2 wild-type strain (*11*, *16*). Recently, we have shown that the expression of *daf-7* in the ASJ neurons is part of a dynamic gene expression feedback loop that promotes increased duration of the roaming state (*16*).

**Fig. 4.**
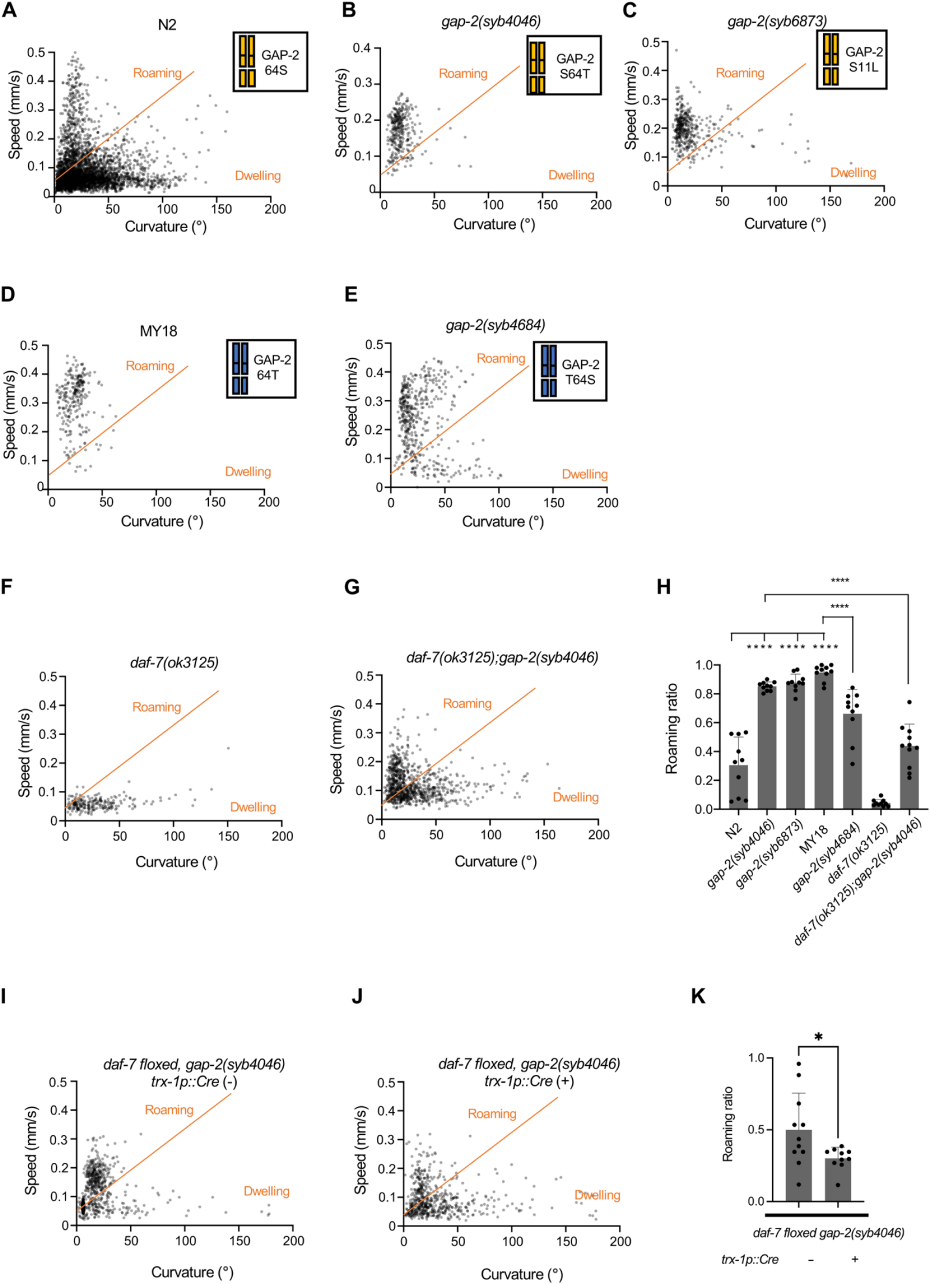
*gap-2* variants affect foraging behavior. (**A** to **I**) Scatter plot of speed and curvature of each indicated strain. Each dot represents average speed and body curvature during 10 s. The orange line was determined on total N2 data and used for analyses of all the strains. Chromosome diagrams indicate *gap-2* allele and genetic background (yellow: N2, blue: MY18). (**J**) Roaming ratio of N2, *gap-2(syb4046)*, *gap-2(syb6873)*, MY18, *gap-2(syb4684)*, *daf-7(ok3125)*, and *daf-7(ok3125); gap-2(syb4046)*. Each dot represents average roaming ratio of one animal. ****P < 0.0001 as determined by an unpaired two-tailed *t* test. (**K**) Roaming ratio of *daf-7* floxed *gap-2(syb4046)* with or without *trx-1p::Cre* transgene expression. Each dot represents average roaming ratio of one animal. *P < 0.05 as determined by an unpaired two-tailed *t* test.

We observed that the MY18 strain exhibited a marked increase in the proportion of time spent in the roaming state relative to the N2 strain (Fig. 4, A, D, and H). The ancestral 215F allele of *npr-1* is known to contribute to increased roaming behavior when compared to the N2 strain that carries a laboratory-derived 215V mutation in *npr-1* (*25*). To examine the effect of the 64T *gap-2* variant on foraging behavior, we observed the strain carrying the *gap-2(syb4046)* 64T allele in the N2 genetic background and found this strain spent an increased proportion of time in the roaming state relative to the N2 strain (Fig. 4, A, B, and H). In addition, we observed that the strain carrying the *gap-2*(*syb4684*) 64S allele in the MY18 genetic background spent a decreased proportion of time in the roaming state (Fig. 4, D, E, and H). These data establish that the 64T variant in *gap-2* promotes an increase in the proportion of time that the animal is in the roaming state relative to the dwelling state when compared with the 64S allele of *gap-2* in the N2 or MY18 genetic backgrounds. We also observed that the strain carrying the *gap-2* allele with the 11L variant, *gap-2(syb6873)*, in the N2 genetic background also increased the proportion of time that animals spent in the roaming state, to a degree comparable to what was observed for the 64T variant in the N2 background (Fig. 4, A, C, and H). We also ruled out that a large defect in feeding and growth might account for the marked effect of the variants on roaming behavior. The *gap-2(syb4046)* and *gap-2(syb6873)* animals exhibited a ~20% decrease in pharyngeal pumping rate and wild-type rates of growth and development (fig. S4). Our data identify two distinct variants acting on the same exon specific to *gap-2g* and *gap-2j* isoforms having comparable effects on *daf-7* ASJ expression and foraging behavior.

### *gap-2* variants causing differences in foraging behavior by modifying *daf-7* neuroendocrine gene expression

To determine whether the *gap-2* variants modified roaming behavior by their effects on *daf-7* ASJ expression, we first examined the roaming and dwelling behavior of the strain carrying the T64 *gap-2* variant and a loss-of-function *daf-7* allele in the N2 genetic background. We observed that this *gap-2(syb4046); daf-7(ok3125)* double mutant exhibited diminished roaming behavior compared with a strain carrying only the *gap-2(syb4046)* allele in the N2 background (Fig. 4, F, G, and H). Next, we examined the roaming behavior of a strain carrying the *gap-2(syb4046)* allele and a floxed *daf-7* locus, with and without a transgene expressing Cre under an ASJ-specific (*trx-1p*) promoter. We observed a diminished proportion of roaming behavior in the animals carrying a selective knockout of *daf-7* from the ASJ neurons (Fig. 4, I to K). These data suggest that the effect of the *gap-2(syb4046)* variant on *daf-7* ASJ expression contributes to the foraging behavior trait difference between the N2 and MY18 strains, although the partial suppression of the increased roaming conferred by the *gap-2(syb4046)* variant suggests that the variant might also act through DAF-7–independent mechanisms to promote roaming behavior.

### *gap-2* variants acting cell nonautonomously to modulate neuroendocrine gene expression and foraging behavior

**Fig. 5.**
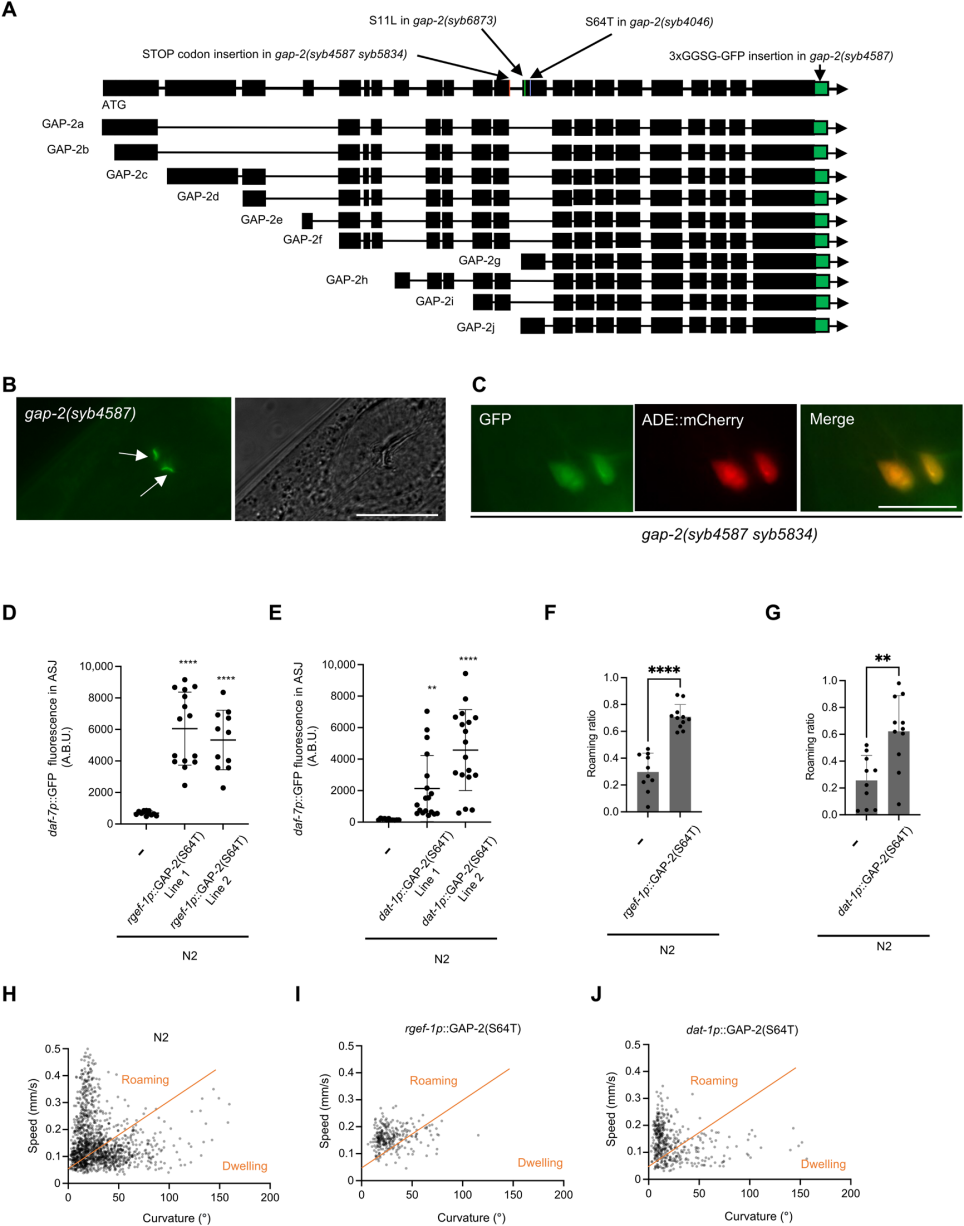
Natural variants in *gap-2* act in the ADE pair of neurons to modulate *daf-7* ASJ expression and foraging behavior. (**A**) Diagram of the *gap-2* genomic locus with C-terminal GFP tagging and stop codon insertion engineered to facilitate the determination of expression pattern. (**B**) GFP tagged GAP-2 G-/J- isoform expression of the *gap-2(syb4587 syb5834)* strain (left) and Nomarski image (right). Scale bar, 10 μm. Arrows point to sensillar endings of ADE neurons. (**C**) Colocalization of GAP-2 G-/J- isoform and *dat-1p*::mCherry reporter in the cell bodies of the ADE neuron pair. Scale bar, 10 μm. (**D**) Maximum fluorescence values *daf-7p*::GFP in the ASJ neurons of pan-neuronal GAP-2(64T) expressing transgenic strains. Each dot represents an individual animal, and error bars indicate SDs. ****P < 0.0001 as determined by an unpaired two-tailed *t* test. Each genotype was compared to wild type (WT). (**E**) Maximum fluorescence values *daf-7p*::GFP in the ASJ neurons of ADE neuronal GAP-2(64T) expressing transgenic strains. Each dot represents an individual animal, and error bars indicate SDs. ****P < 0.0001 and **P < 0.01 as determined by an unpaired two-tailed *t* test. Each genotype was compared to WT. (**F**) Roaming ratios of pan-neuronal GAP-2(64T) expressing transgenic strains. Each dot represents an individual animal, and error bars indicate SDs. ****P < 0.0001 as determined by an unpaired two-tailed *t* test. (**G**) Roaming ratios of ADE neuronal GAP-2(64T) expressing transgenic strain. Each dot represents an individual animal, and error bars indicate SDs. ****P < 0.0001 as determined by an unpaired two-tailed *t* test. (**H** to **J**) Scatter plot of speed and curvature of each indicated strain. Each dot represents average speed and body curvature during 10 s. Orange line has been determined on total N2 data and used for analysis of all the strains.

To determine the site of action where *gap-2* variants modify neuroendocrine gene expression and foraging behavior, we first defined the expression pattern of *gap-2* by green fluorescent protein (GFP)–tagging endogenous GAP-2 at its C terminus. We observed expression in multiple cells of the nervous system (fig. S4), consistent with prior reports of the tissue expression pattern of *gap-2* (*26*). To determine the cells in which the *g-* and *j-* isoforms of *gap-2* were expressed, we inserted a stop codon in the exon preceding the first exon of the *g-* and *j-* isoforms, which was expected to cause only translation of GFP-tagged G- and J- isoforms of GAP-2 (Fig. 5, A and B). This strain exhibited GFP expression in the sensillar endings of the ADE neuron pair based on cell location and morphology (Fig. 5B). We confirmed expression of the G- and J- isoforms of GFP-tagged GAP-2 in the ADE neuron pair through colocalization with the expression of the *dat-1p::mCherry* transgene that was expressed in the ADE neuron pair (Fig. 5C).

We used the expression pattern information to determine the neurons in which expression of the T64 variant of GAP-2 was sufficient to confer increased *daf-7* ASJ expression and roaming behavior. Because the 64T variant causes a dominant effect on *daf-7* expression, we used a transgene expressing the *gap-2g*-isoform cDNA carrying the 64T variant under the control of the pan-neuronal *rgef-1* promoter in the N2 genetic background. We also expressed the *gap-2g*-isoform cDNA carrying the 64T variant under the control of the ADE neuronal *dat-1* promoter in the N2 background. We observed that the expression of the 64T GAP-2 sequence under the control of both pan-neuronal and ADE neuronal promoters, in the N2 genetic background, conferred *daf-*7 ASJ expression (Fig. 5, D and E) and an increased proportion of time spent in the roaming state (Fig. 5, F to J). These data suggested that expression of the GAP-2 variant in the ADE neurons was sufficient to act cell nonautonomously to alter the expression of *daf-7* from the ASJ neurons and to modify foraging behavior.

### Prevalence and geographical distribution of *gap-2* variants

**Fig. 6.**
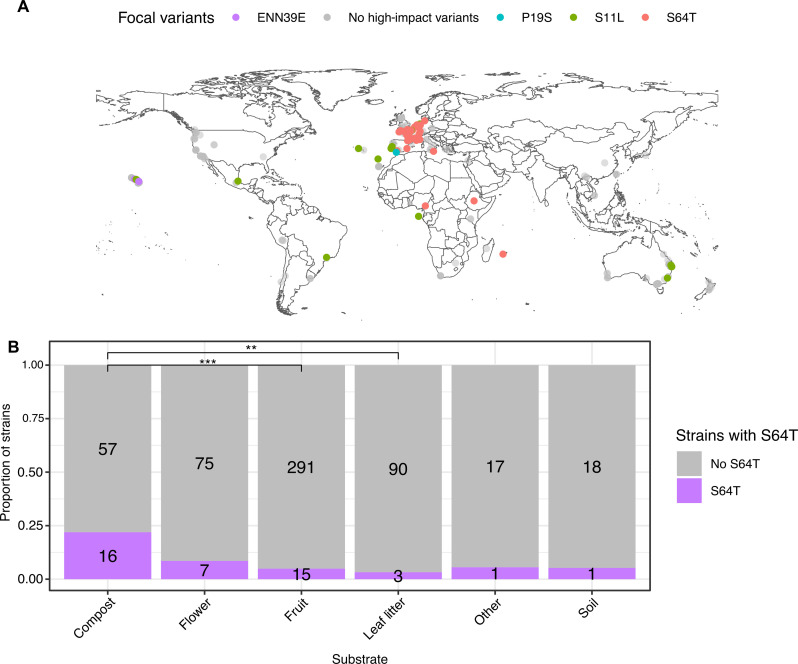
Environmental enrichment of the *gap-2* 64T allele is observed in natural populations. (**A**) *gap-2* haplotypes of wild *C. elegans* strains are represented by colored shapes (32 unique haplotypes across 550 strains) and are plotted by their sampling location. Shapes distinguish the presence or absence of a naturally occurring variant predicted to affect the splicing or protein coding sequence of *gap-2* g/j. (**B**) Substrate enrichment of the S64T across wild strains. A Fisher’s exact test was used to evaluate significance. ***P < 0.001, **P < 0.01.

The 64T *gap-2* variant is present in 48 of 550 isotype reference strains in the *Caenorhabditis* Natural Diversity Resource (*22*), whereas the 11L *gap-2* variant is found in 24 of these strains. We also identified two additional rare variants, ENN39E and P19S, which are also predicted to affect the *g-* and *j-* isoforms of *gap-2*. We examined the geographic origins of these 72 wild strains and observed enrichment for the T64 allele in Africa and Europe (Fig. 6A). We found that the 11L and ENN39E variants have also been observed in divergent strains from the Hawaiian Islands. Because *daf-7* expression is affected by the bacterial diet, we investigated enrichment in specific natural substrates and found that the T64 variant was more often found in strains isolated from compost as compared strains isolated from rotting fruit or leaf litter (Fig. 6B).

## DISCUSSION

Our data illustrate how changes in neuronal gene expression caused by natural variants underlie the mechanism by which the variants can exert differential effects on behavioral traits. In particular, we show that natural variants cause differences in behavior by cell-nonautonomously modulating expression levels of a neuroendocrine regulatory ligand in two neurons of *C. elegans*. The systematic analysis of gene expression as a quantitative trait has been generally conducted on a genome-wide scale enabled by RNA sequencing–based methodology (*8*). Our focus on the expression of a single gene enabled us to use a transgenic fluorescent reporter to examine levels of neuron-specific expression in live animals and facilitated subsequent mapping and molecular characterization. Roles for neuromodulators in shaping circuits that govern behavior have been implicated from genetic studies (*27*, *28*), and our data suggest that variants causing differences in expression levels of genes that encode neuromodulators represent candidate variants that cause differences in behavior. In the current study, our focus on natural variation in *daf-7* expression from the ASJ neurons was also motivated by our prior work establishing that *daf-7* ASJ expression levels are strongly correlated with internal state favoring exploratory behavior (*16*).

GAP-2 is one of three GAPs in the *C. elegans* genome that stimulate the LET-60 Ras GTPase to reduce epidermal growth factor signaling (*26*, *29*). GAP-2 is orthologous to disabled homolog 2-interacting protein (DAB2IP), a member of the SynGAP family of GAPs, which has been implicated in a range of disease states (*30*, *31*) The similar dominant effects on *daf-7* ASJ expression for both of the 64T and 11L natural variants as compared to the recessive effects of a deletion of *gap-2* suggest that 64T and 11L variants confer reduced GAP-2 activity. We speculate that the 64T and 11L GAP-2G and/or GAP-2J isoforms act as dominant-negative proteins that might bind to LET-60 but not activate the GTPase and compete with wild-type GAP-2. Both *let-60(n1046)* gain-of-function and *let-60(n2021)* reduction-of-function alleles exhibit pronounced locomotory phenotypes in the presence of bacterial food (*32*), precluding the analysis of genetic interactions between *gap-2* and *let-60*, but the presence of locomotory defects is consistent with *gap-2* affecting Ras signaling in the ADE neurons to alter foraging behavior. Alterations in Ras signaling in the ADE neurons may also modify dopamine-dependent or PDF-1–dependent signaling that have been shown to have activity in the ADE neurons (*33*, *34*), which may act cell nonautonomously to modulate *daf-7* ASJ expression in the ASJ neurons. Our data also illustrate how the presence of multiple alternatively spliced transcripts of *gap-2* might facilitate the emergence of genetic variants that exert effects in a restricted set of neurons. Whereas GAP-2 appears to be widely expressed in the nervous system and in other tissues, the GAP-G and/or GAP-2J isoforms exhibit restricted expression in the ADE pair of sensory neurons, and two variants, both 64T and 11L, affect an exon specific to these two isoforms.

The genetic variants in *gap-2* that modulate *daf-7* expression from the ASJ neurons and promote roaming behavior are found in many strains throughout the *C. elegans* species, suggesting that the variants may confer a fitness advantage in diverse bacterial environments. The enrichment of these *gap-2* variants in strains isolated from compost relative to strains isolated from rotting fruit or leaf litter substrates is intriguing because *C. elegans* isolated from compost are more commonly found as dauer larvae (*35*). We speculate that animals carrying *gap-2* variants conferring increased exploratory behavior might gain an advantage in compost environments that are enriched for dauer larvae. Our data suggest that diversity in behavioral traits associated with host interactions with microbes (*36*) can emerge from variants affecting the regulation of neuronal gene expression that is modulated by bacteria-derived food and pathogen cues.

## MATERIALS AND METHODS

### *C. elegans* culture

All strains were grown at 20°C on nematode growth medium plates seeded with *E. coli* OP50 bacteria. OP50 was inoculated into 50 ml of Luria Broth (LB) broth and grown 24 hours at 37°C. See table S2 for a complete list of strains used in this study. Sequence information of strains generated by CRISPR-Cas9 genome editing is in table S3.

### *daf-7p*::GFP expressing wild isolate strain construction

To generate *daf-7p*::GFP carrying natural isolate reporter strains, each wild strain of interest was initially crossed to FK181 to introduce the *ksIs2* transgene. Progeny carrying the transgene were then backcrossed to the parental wild strain eight times.

### NIL construction

ZD1271 was made by backcrossing the ZD841 eight times to FK181 by following the *daf-7p*::GFP expression in ASJ phenotype. ZD2589 was made by crossing the ZD1271 with FK181 and then screening the strains for having N2 genotype region in between the Chr. X 9.5 to 14 Mb. ZD2626 was made by crossing ZD2589 with FK181 and then screening the strains for having N2 genotype in between Chr. X 9.5 to 12 Mb. ZD2627 was made by crossing ZD2589 with FK181 and then screening the strains for having N2 genotype in Chr. X 1.7 to 8.9 Mb. ZD2671 was made by crossing ZD2589 with FK181 and then screening the strains for having N2 genotype in between Chr. X 9.5 to 10 Mb.

### Linkage mapping

*daf-7p*::GFP expression in RILs were measured, and whole-genome sequencing results of each RIL strain were used for linkage mapping. A total of 123 RILs were examined for the level of *daf-7p*::GFP expression in ASJ neurons as described above. Linkage mapping was performed for *daf-7p*::GFP expression in ASJ neurons using R package linkage mapping (www.github.com/AndersenLab/linkagemapping as previously described (*37*). QTLs were detected using the fsearch function. This function calculates the logarithm of the odds (LOD) scores for each genetic marker and each trait as −*n*[ln(1 − *R*^2^)/2ln(10)] where *R* is the Pearson correlation coefficient between the RIL genotypes at the marker and trait values (*38*). A threshold for significance based on a 5% genome-wide error rate was calculated. QTLs were identified as the marker with the highest LOD score above the significance threshold. This marker was then integrated into the model as a cofactor, and mapping was repeated until no significant QTLs were detected. The annotated lods function was used to calculate the effect size of each QTL. Confidence intervals (95%) were defined by a 1.5-LOD drop from the peak marker.

### *daf-7p*::GFP expression imaging

For quantification of *daf-7* expression in animals, day 1 young adult animals were mounted and anesthetized in levamisole. The animals were imaged at 40× using a Zeiss Axio Imager Z1 microscope. Fifteen to 20 animals were imaged for each condition or strain. For quantification, maximum intensity values of GFP within the ASJ neurons were calculated using FIJI software (*39*).

### Roaming/dwelling assay

Day 1 adults were placed on 10-cm Nematode Growth Medium (NGM) plates, on which fresh OP50 had been uniformly spread the day before the experiment. These plates contained a 6-cm copper ring to keep the animals within the field of view for recording. The worms were transferred to inside of the ring on the assay plates 40 min before to move to worm tracker to avoid the initial reaction of worms to picking and transfer. On average, 10 worms were recorded. We recorded the region inside the copper ring at 3.75 frames per second for 90 min. Videos were analyzed using MBF Biosciences WormLab software. Speed and mid body bending angle were averaged over 10-s intervals. Values for each 10-s interval were plotted on a scatter plot of speed (*y* axis) and bending angle (*x* axis). Quantification of fraction of time spent roaming was done by the diagonal line whose placement was based on the distribution of points of the distribution of N2. Equation for this line is *y* = 0.3*x + 5. Points falling above the line were classified as roaming, and those points below the line were classified as dwelling.

### Statistics

All statistical analysis was performed using the GraphPad Prism software. Statistical tests used for each experiment are listed in the figure legend.

## Supplementary Materials

### This PDF file includes:

Figs. S1 to S4 Tables S1 to S3 References
