# Supplementary material for: Genetic variants that modify neuroendocrine gene expression and foraging behavior of C. elegans
Source: Sci Adv. 2024 Jun 12;10(24):eadk9481. doi: 10.1126/sciadv.adk9481 (PMC11168454; doi:10.1126/sciadv.adk9481)
Supplement: Supplementary file 1 — Figs. S1 to S4 Tables S1 to S3 References [file sciadv.adk9481_sm.pdf]

Science Advances
AAAS

Supplementary Materials for

Genetic variants that modify neuroendocrine gene expression and foraging behavior of *C. elegans*

Harksun Lee *et al.*

Corresponding author: Dennis H. Kim, dennis.kim@childrens.harvard.edu

Sci. Adv. **10**, eadk9481 (2024)
DOI: 10.1126/sciadv.adk9481

This PDF file includes:

Figs. S1 to S4
Tables S1 to S3
References

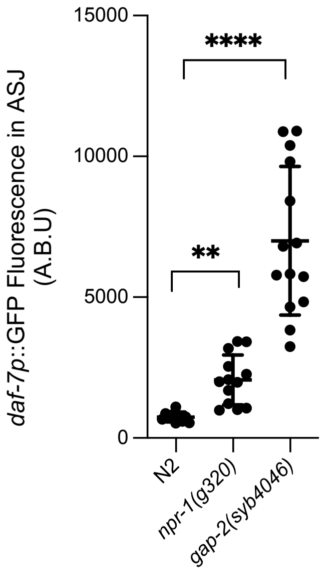

Fig. S1. NPR-1(215F) affects *daf-7* expression in the ASJ neurons. Maximum fluorescence values of *daf-7p*::GFP in the ASJ neurons in *npr-1(g320)* and *gap-2(syb4046)*. Each dot represents an individual animal, and error bars indicate standard deviations. ****p<0.0001 and **p<0.01 as determined by an unpaired two-tailed t-test compared to N2.

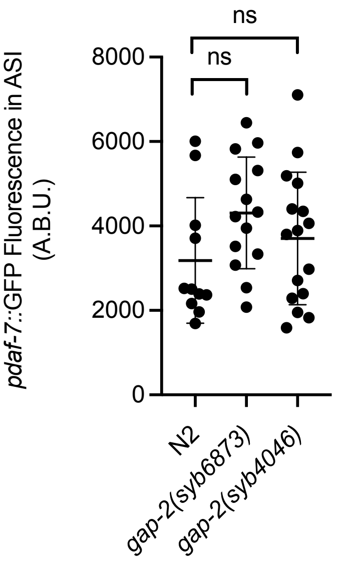

Fig. S2. GAP-2(S64T) or GAP-2(S11L) variant does not affect the *daf-7* expression in ASI neurons. Maximum fluorescence values *daf-7p*::GFP in the ASI neurons of indicated strains. Each dot represents an individual animal, and error bars indicate standard deviations. "ns" as determined by an unpaired two-tailed t-test. Each genotype was compared to N2.

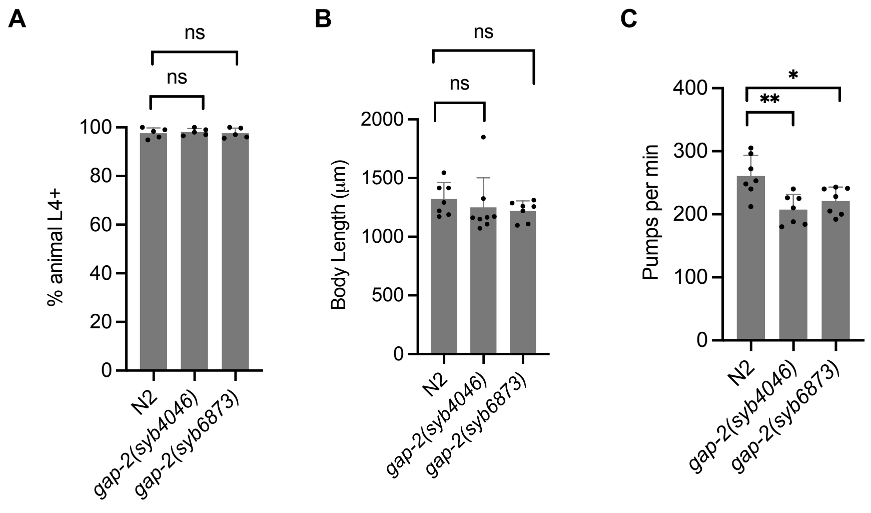

Fig. S3. GAP-2(S64T) or GAP-2(S11L) variant does not affect the growth rate and body size but affects the pumping rate. (**A**) % animal L4+ animals 49hr after egg laying. n>100, 3 biological replicate at each strain. “ns” as determined by an unpaired two-tailed t-test. (**B**) Length of the body 24 hours after L4 stage. Each dot represents body length of one animal. “ns” as determined by an unpaired two-tailed t-test. (**C**) The pumping rate of young adult worms. Each dot represents number of pumps per min of one animal. *$p<0.05$ and **$p<0.01$ as determined by an unpaired two-tailed t-test.

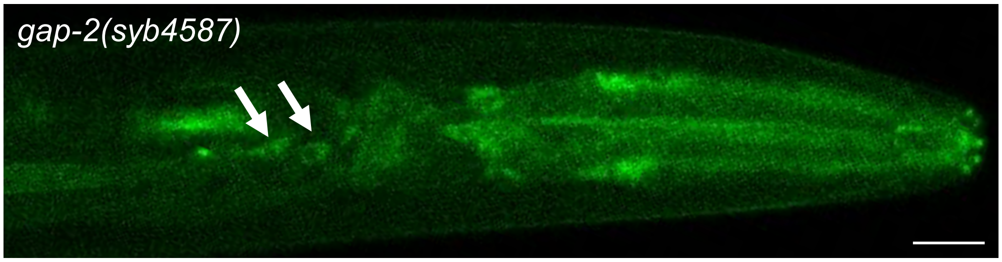

Fig. S4. GFP tagged GAP-2 expression of the *gap-2(syb4587)* strain. Scale bar, 10 μm. Arrows point to ADE neurons.

Strain	P value
KR314	<0.0001
JU1088	0.0012
ED3040	<0.0001
RC301	0.0008
CB4856	<0.0001
JU1400	<0.0001
JU394	<0.0001
AB4	<0.0001
ED3005	0.0004
JU775	<0.0001
MY16	<0.0001
ED3017	0.0035
MY1	<0.0001
CB4854	<0.0001
JU778	<0.0001
JU258	<0.0001
CB4932	<0.0001
PX174	<0.0001
PB306	<0.0001
MY14	<0.0001
JU346	<0.0001
ED3011	<0.0001
JU406	<0.0001
JU1440	<0.0001
MY18	<0.0001
ED3046	<0.0001
JU1401	<0.0001
JU397	<0.0001
ED3052	<0.0001

Table S1. Statistical test result for *daf-7* expression of wild isolates (Figure 1B). P values were determined by an unpaired two-tailed t-test compared to N2.

Strain Name	Description	Source
N2	Wild type	Caenorhabditis Genetics Center (CGC)
FK181	*ksIs2[pdaf-7::gfp;rol-6(su1006)]*	CGC
ZD1190	KR314 crossed FK181 for 8 times to introduce *ksIs2*	This study
ZD838	JU1088 crossed FK181 for 8 times to introduce *ksIs2*	This study
ZD1097	ED3040 crossed FK181 for 8 times to introduce *ksIs2*	This study
ZD1075	RC301 crossed FK181 for 8 times to introduce *ksIs2*	This study
ZD710	CB4856 crossed FK181 for 8 times to introduce *ksIs2*	This study
ZD1635	JU1400 crossed FK181 for 8 times to introduce *ksIs2*	This study
ZD1028	JU394 crossed FK181 for 8 times to introduce *ksIs2*	This study
ZD837	AB4 crossed FK181 for 8 times to introduce *ksIs2*	This study
ZD1120	ED3005 crossed FK181 for 8 times to introduce *ksIs2*	This study
ZD834	JU775 crossed FK181 for 8 times to introduce *ksIs2*	This study
ZD840	MY16 crossed FK181 for 8 times to introduce *ksIs2*	This study
ZD1073	ED3017 crossed FK181 for 8 times to introduce *ksIs2*	This study
ZD1187	MY1 crossed FK181 for 8 times to introduce *ksIs2*	This study
ZD1119	CB4854 crossed FK181 for 8 times to introduce *ksIs2*	This study
ZD839	JU778 crossed FK181 for 8 times to introduce *ksIs2*	This study
ZD1096	JU258 crossed FK181 for 8 times to introduce *ksIs2*	This study
ZD1638	CB4932 crossed FK181 for 8 times to introduce *ksIs2*	This study
ZD1188	PX174 crossed FK181 for 8 times to introduce *ksIs2*	This study
ZD1027	PB306 crossed FK181 for 8 times to introduce *ksIs2*	This study
ZD1189	MY14 crossed FK181 for 8 times to introduce *ksIs2*	This study
ZD1026	JU346 crossed FK181 for 8 times to introduce *ksIs2*	This study
ZD1025	ED3011 crossed FK181 for 8 times to introduce *ksIs2*	This study
ZD1636	JU406 crossed FK181 for 8 times to introduce *ksIs2*	This study
ZD1074	JU1440 crossed FK181 for 8 times to introduce *ksIs2*	This study
ZD841	MY18 crossed FK181 for 8 times to introduce *ksIs2*	This study
ZD1633	ED3046 crossed FK181 for 8 times to introduce *ksIs2*	This study
ZD1186	JU1401 crossed FK181 for 8 times to introduce *ksIs2*	This study

ZD1632	JU397 crossed FK181 for 8 times to introduce *ksIs2*	This study
ZD1637	ED3052 crossed FK181 for 8 times to introduce *ksIs2*	This study
ZD1271	Near Isogenic Line (MY18 region Chr. X 1.7~8.9Mb and Chr. X 9.5Mb~14Mb)	This study
ZD2589	Near Isogenic Line (MY18 region Chr. X 1.7~8.9Mb and Chr. X 9.5Mb~ 12Mb)	This study
ZD2626	Near Isogenic Line (MY18 region Chr. X 1.7~8.9Mb and Chr. X 9.5 ~ +10Mb)	This study
ZD2627	Near Isogenic Line (MY18 region Chr. X 9.5Mb~ 10Mb)	This study
ZD2671	Near Isogenic Line (MY18 region Chr. X 9.5Mb~ 9.9Mb)	This study
ZD2683	*ksIs2;gap-2(syb4046)*	This study/SunyBiotech
PHX4046	*gap-2(syb4046)*	This study/SunyBiotech
PHX5109	*gap-2(syb5109)*	This study/SunyBiotech
PHX5128	*gap-2(syb5128)*	This study/SunyBiotech
PHX6873	*gap-2(syb6873)*	This study/SunyBiotech
PHX4684	*gap-2(syb4684)*	This study/SunyBiotech
PHX4587	*gap-2(syb4587)*	This study/SunyBiotech
PHX5834	*gap-2(syb4587 syb5834)*	This study/SunyBiotech
JN147	*gap-2(tm748)*	National BioResource Project (NBRP)
RB2302	*daf-7(ok3125)*	CGC
ZD2687	*ksIs2;gap-2(tm748)*	This study
ZD2704	N2;Ex[*rgef-1p*::GAP-2(S64T) + PQZ22]	This study
ZD2705	N2;Ex[*ceh-2p*::GAP-2(S64T) + PQZ22]	This study
ZD2706	N2;Ex[*trx-1p*::GAP-2(S64T) + PQZ22]	This study
ZD2707	FK181;Ex[*rgef-1p*::GAP-2(S64T) + PQZ22]	This study
ZD2708	FK181;Ex[*ceh-2p*::GAP-2(S64T) + PQZ22]	This study
ZD2709	FK181;Ex[*trx-1p*::GAP-2(S64T) + PQZ22]	This study
ZD2713	N2;EX[*dat-1p*::GAP-2(S64T) + PQZ22] TG line 1	This study
ZD2714	N2;EX[*dat-1p*::GAP-2(S64T) + PQZ22] TG line 2	This study
ZD2715	N2;EX[*dat-1p*::GAP-2(S64T) + PQZ22] TG line 3	This study
ZD2716	FK181;Ex[*dat-1p*::GAP-2(S64T) +PQZ22] TG line 1	This study
ZD2717	FK181;Ex[*dat-1p*::GAP-2(S64T) +PQZ22] TG line 2	This study

Table S2. Complete list of *C. elegans* strains used in this study.

wild type	*gap-2(syb4046)*
taagcccatcgtctcgtgtcgttctgatgactcaat<br>ctctgactagccaacctttttcactttttttcgcactca<br>attctaaattctctctatttcttgtatcactttttttg<br>tgtagcaatcaaccattatccacgccttcagattgtc<br>ATGAGAGGTGAATACGATCCATGGGATCCATCGTTTC<br>ACAATTCTTCTATGATTCCGTATTCCCTTGCTTCCAT<br>ATATCCGTCAATCGAAAATTTGCCAGAGGAATTTTCA<br>AATGAAAATAATAAAATTAAAAATGTCTTGAAAAACT<br>TTATTTGGTCATTCAAGCTCAAGAAAAATTACGTGCG<br>GGTATCGTCGTTGTTCGGAGAgtattgtaggtcagta<br>gtaaattagattatcaaaatcccatacagtacaggtc<br>gccatcaaggttacaccccgacagtataagttaatcc<br>gcgttttcgattccagGTATACAGTCAACACATCTCA<br>TAGTGACTCTGGGACAAGCAGAATTGCATCCGCACTA<br>GGTGGGAAGAGCAGCTCCCAGGAATCCCCATCGCTTA<br>GAATCAAAGCCCGTTGGCAGTCGGTGCACATCCTCCC<br>ACTTCGAGCCTACGACAACCTTCTGGAAACACTTTGC<br>TATAACTATTTGCCGCTTTGCGAGCAATTGGAGCCAG<br>TGCTCAATGTCAGAGACAAGgtaaacaaaattaatct<br>agaatagcccgagatttgaaattttcacacatgaaca<br>tagtttcagGAGGACTTGGCGACATCGTT	taagcccatcgtctcgtgtcgttctgatgactcaatc<br>tctgactagccaacctttttcactttttttcgcactcaa<br>ttctaaattctctctatttcttgtatcactttttttgt<br>gtagcaatcaaccattatccacgccttcagattgtcA<br>TGAGAGGTGAATACGATCCATGGGATCCATCGTTTCA<br>CAATTCTTCTATGATTCCGTATTCCCTTGCTTCCATA<br>TATCCGTCAATCGAAAATTTGCCAGAGGAATTTTCAA<br>ATGAAAATAATAAAATTAAAAATGTCTTGAAAAACTT<br>TATTTGGTCATTCAAGCTCAAGAAAAATTACGTGCGG<br>GTAACAAGCTTGTTCGGAGAgtattgtaggtcagtag<br>taaattagattatcaaaatcccatacagtacaggtcg<br>ccatcaaggttacaccccgacagtataagttaatccg<br>cgttttcgattccagGTATACAGTCAACACATCTCAT<br>AGTGACTCTGGGACAAGCAGAATTGCATCCGCACTAG<br>GTGGGAAGAGCAGCTCCCAGGAATCCCCATCGCTTAG<br>AATCAAAGCCCGTTGGCAGTCGGTGCACATCCTCCCA<br>CTTCGAGCCTACGACAACCTTCTGGAAACACTTTGCT<br>ATAACTATTTGCCGCTTTGCGAGCAATTGGAGCCAGT<br>GCTCAATGTCAGAGACAAGgtaaacaaaattaatcta<br>gaatagcccgagatttgaaattttcacacatgaacat<br>agtttcagGAGGACTTGGCGACATCGTT
Background sequence	*syb5109*(-3071bp deletion)
tcaaacattggtcggctcatgatgtgttcccattgat<br>attacgtagtctatgaaattatttcctcttgcgtaat<br>aatggtaatatttcgaaacatgttatttgtacttaga<br>tttcaactttaaatgcaaaccattctttaaaataaaa<br>acttgtcttacgttgttatgaaattgaattattaaaa<br>aaatctttggcccttagattaggttttttttaattgt<br>aatttttatcaatgtactctcccagttattcctagtt<br>tttcaatttaatttagtaactgacatgttattgaatt<br>gattgcaatgtttctccattcacctgcatttcaatca<br>ctgatggcactattaatgtgagggccaatagtagaac<br>agatcttatctacgctcatagattttaattcccaatt<br>agataagcccatcgtctcgtgtcgttctgatgactca<br>atctctgactagccaacctttttcactttttttcgcact<br>caattctaaattctctctatttcttgtatcactttttt<br>tgtgtagcaatcaaccattatccacgccttcagattg<br>tcATGAGAGGTGAATACGATCCATGGGATCCATCGTT<br>TCACAATTCTTCTATGATTCCGTATTCCCTTGCTTCC<br>ATATATCCGTCAATCGAAAATTTGCCAGAGGAATTTT<br>CAAATGAAAATAATAAAATTAAAAATGTCTTGAAAAA<br>CTTTATTTGGTCATTCAAGCTCAAGAAAAATTACGTG<br>CGGGTAACAAGCTTGTTCGGAGAgtattgtaggtcag<br>tagtaaattagattatcaaaatcccatacagtacagg<br>tcgccatcaaggttacaccccgacagtataagttaat<br>ccgcgttttcgattccagGTATACAGTCAACACATCT<br>CATAGTGACTCTGGGACAAGCAGAATTGCATCCGCAC<br>TAGGTGGGAAGAGCAGCTCCCAGGAATCCCCATCGCT<br>TAGAATCAAAGCCCGTTGGCAGTCGGTGCACATCCTC<br>CCACTTCGAGCCTACGACAACCTTCTGGAAACACTTT<br>GCTATAACTATTTGCCGCTTTGCGAGCAATTGGAGCC<br>AGTGCTCAATGTCAGAGACAAGgtaaacaaaattaat<br>ctagaatagcccgagatttgaaattttcacacatgaa<br>catagtttcagGAGGACTTGGCGACATCGTTGGTTCG	Tcaaacattggtcggctcatgatgtgttcccattgat<br>attacgtagtctatgaaattatttcctcttgcgtaat<br>aatggtaatatttcgaaacatgttatttgtacttaga<br>tttcaactttaaatgcaaaccattctttaaaataaaa<br>acttgtcttacgttgttatgaaattgaattattaaaa<br>aaatctttggcccttagattaggttttttttaattgt<br>aatttttatcaatgtactctcccagttattcctagtt<br>tttcaatttaatttagtaactgacatgttattgaatt<br>gattgcaatgtttctccattcacctgcatttcaatca<br>ctgatggcactattaatgtgagggccaatagtagaac<br>agatcttatctacgctcatagattttaattcccaatt<br>agataagcccatcgtctcgtgtcgttctgatgactca<br>atctctgactagccaacctttttcactttttttcgcact<br>caattctaaattctctctatttcttgtatcactttttt<br>tgtgtagcaatcaaccattatccacgccttcagattg<br>tc-<br>tcaatatcaaatattatcctacagttattagtcttat<br>tattattttacctatttatttttatcgatctattggt<br>tcttcttgtcttaatgtaactaatgatgttcccaaag<br>aaataaaagtacatatactgtcttcccctgcactccc<br>gttggtctcttcatttgcctgccgccaacctttcgat<br>tcagtacacatcccttatctaaaggcttttcatgcat<br>attttgaatatctaaagttctcctgctcacctgtcat<br>tttttgtagtttagagggtttttgattgtgattttttt<br>aattttcaatatatgtcttcttccctccactacccat<br>cttccgatgtacaacaacgtaaactttgtcgcactgc<br>ttgtttttttagctccatacactggagatatgaaaact<br>acccggtagcggtgttgttagccaa

TGTTATGTACAAACACAACCTCGCAAAGGAGTTCCTG<br>TGTGATTTGATCATGAAGGAGGTCGAGAAGCTCGACA<br>ATGATCATTTAATGTTCAGAGGAAACACACTGGCCAC<br>AAAGGCTATGGAGTCGTTTATGAAACTTGTCGCCGAC<br>GATTATCTAGACTCAACACTCAGTGATTTTATTAAAA<br>CAGTGTTACAATGTGAGGATTCATGCGAAGTAGATCC<br>ACAGAAATTGGGTAATGTGTCAAACTCATCCCTCGAG<br>AAGAATCGTGCCCTTTTGATGCGATATGTTGAAGTGG<br>CTTGGACGAAAATTTTGAACAAgtatgttgcatgcta<br>atttcacttatgtttttatccattgaattttagCGTT<br>CACCAGCTACCAAAAAACCTCCGAGACGTATTTTCGG<br>CTCTTCGTTGCCGACTTGAAGCCCAGAATCGTGAAGC<br>TTTGGCAGATACTTTGATCTCCTCATCAATCTTCCTT<br>CGATTCTTATGTCCTGCAATTTTGAGCCCAAGCCTTT<br>TCAATCTTGTTTCCGAGTACCCATCACCAACGAACGC<br>GCGCAACTTGACTCTGATTGCCAAGACGCTGCAGAAC<br>CTTGCGAATTTCAGCAAGTTTGGAGGAAAAGAGCCTC<br>ACATGGAGTTTATGAACGAGTTTGTCGACCGAGAGTG<br>GCATAGGATGAAAGATTTCTTGTTGAGAATTTCTTCA<br>GAgtgagttgacgatgtatcacgttacataggtgcag<br>tattttgaagtactctatgtgctaaatttggcactat<br>tattaaataaatagagtataaaaatagaataacacac<br>tcacactcattgcaaagatcaaaacaattaatgacaa<br>ttttcagGTCAAAGTCGGGACCAGAAAAAAATGCGGA<br>TGCAATTGTGGACGCCGGCAAAGAGCTTAGCTTAATC<br>GCTACTTATCTTGAAGAAGCCTGGACTCCACTTCTCC<br>AAGAGAAAAATGGAAATAAGCATCCACTCTCAAATGT<br>CAAGTCGGTTTTATCCGAATTGGCAGAATGCAAGAGA<br>CGCTCGGATAATGGCGTCTTTCACTCTCCAATGGTTC<br>AACAGCCGTCTTCAGATTATGAGAATAGTCCACAGCA<br>ACACGTTGTgtgagtttcgtcttccgactttcgagtt<br>ccgagtttttgcacgcacccaccgtcattcttaacac<br>aaactaccggtcttttgtttctttaaccattatgtta<br>atacgtgttttttttcttaataaaaatttttgtctat<br>tgtagTCCTCGACATGAGAATGTACCGGCATATCGCA<br>GTACTCCGCCAACTGGCCAAGCCACGGTATTGGGCCG<br>TTCTACAAACCGCCCTGCAACTCATTTGCTCACATCG<br>GACGATTACGTCCTCTCTTCTGCATTTCAAACTCCAA<br>GTCTTCGCCCTGGAGGCACTCGGTTAAGCGATGAAAC<br>TGGCACGTCTTCGAGTCGCACCAGTGACAAGACAACC<br>AGCAGCGCTGAGATTCGAGACGACACTGATTCGGATT<br>TCGAGTTACGAGAGGATCGAGGACGTGGAGGAAGAAA<br>CCGTAAGAGGCTACCACGTACTGATGCATCACCATCG<br>AGCAGTCAACAAGCTTCAAGTGGATATTTAAGTAATA<br>ATCCTTCGAGgtaagatcacatttttctagttcaggc<br>ttctaattcggtatatgatatttcagATCCAGCTACT<br>CAAACTCTTCGAGTTCATCTCCAGTTGAACGAATGGC<br>CGCTCTATCAATTGCTAACCCAGTCTTTGGACCAGGC<br>CCATCATCTGGATATGCTATACCTGCAGAGCCAAAGG<br>AAATCGTATACCAAAAGCGAGCAAGCCCACCACCATA<br>CGATCCCGATGTGCACAACTATCATTATCAACCGgta<br>tgaattgttttcttgtattaaaaatacgttgttattt<br>ttagATGCAGGTCTACGCTGTTCCACCAGATTGTCAG<br>GTGTCCCCAAGAACGCAGGCAACAGGCGGTGTCAATG<br>CTCAGAATCGGTTAAGTCTGCCACGGACTAATCCACG<br>AGCTTCGAGGAATTCAACACTTTTGCGTCCGAGTGTC<br>GTAAATGTTCCGGATGACTGGGATAGgttatttttag<br>aggttattttaaaatctaaattaatgattttcagAAC	

AAGTGATTACTGGAGGGACCGAGGCGAGAACAACTAC<br>CGGAGTCAACTGGAAAGCCAAGTGGAAAGTCAAGCTC<br>GAGAAATTGAACGCTTGATGAGAGAGAACATTGAGCT<br>GAAGAGTAAAATGATGTCTTCAACAAAAACTGTGGAT<br>TCCAAGgtgaattttagaaaatcaaattttgattttt<br>tcaaccaaaaatagtttcagCGATCTGACAGTGGTGC<br>TAGTGAGGATTCCTACGATTCTTTGAGTTCACTCGAT<br>CGTCCATCAAGACAATCGCTTGTGGTAGTTCCAAACT<br>AAtcaatatcaaatattatcctacagttattagtctt<br>attattatttacctatttatttttatcgatctattg<br>gttcttcttgtcttaatgtaactaatgatgttcccaa<br>agaaataaaagtacatatactgtcttcccctgcactc<br>ccgttggtctcttcatttgcctgccgccaacctttcg<br>attcagtacacatcccttatctaaaggcttttcatgc<br>atattttgaatatctaaagttctcctgctcacctgtc<br>atttttttgtagtttagagggtttttgattgtgatttt<br>ttaattttcaatatatgtcttcttccctccactaccc<br>atcttccgatgtacaacaacgtaaactttgtcgcact<br>gcttgtttttttagctccatacactggagatatgaaaa<br>ctacccggtagcggtgttgttagccaa	
Background sequence	*syb5128*(-206bp deletion)
tcaaacattggtcggctcatgatgtgttcccattgat<br>attacgtagtctatgaaattatttcctcttgcgtaat<br>aatggtaatatttcgaaacatgttatttgtacttaga<br>tttcaactttaaatgcaaaccattctttaaaataaaa<br>acttgtcttacgttgttatgaaattgaattattaaaa<br>aaatctttggcccttagattaggtttttttttaattgt<br>aatttttatcaatgtactctcccagttattcctagtt<br>tttcaatttaatttagtaactgacatgttattgaatt<br>gattgcaatgtttctccattcacctgcatttcaatca<br>ctgatggcactattaatgtgagggccaatagtagaac<br>agatcttatctacgctcatagattttaattcccaatt<br>agataagcccatcgtctcgtgtcgttctgatgactca<br>atctctgactagccaacctttttcactttttttcgcact<br>caattctaaattctctctatttcttgtatcactttttt<br>tgtgtagcaatcaaccattatccacgccttcagattg<br>tcATGAGAGGTGAATACGATCCATGGGATCCATCGTT<br>TCACAATTCTTCTATGATTCCGTATTCCCTTGCTTCC<br>ATATATCCGTCAATCGAAAATTTGCCAGAGGAATTTT<br>CAAATGAAAATAATAAAATTAAAAATGTCTTGAAAAA<br>CTTTATTTGGTCATTCAAGCTCAAGAAAAATTACGTG<br>CGGGTAACAAGCTTGTTCGGAGAgtattgtaggtcag<br>tagtaaattagattatcaaaatcccatacagtacagg<br>tcgccatcaaggttacaccccgacagtataagttaat<br>ccgcgttttcgattccagGTATACAGTCAACACATCT<br>CATAGTGACTCTGGGACAAGCAGAATTGCATCCGCAC<br>TAGGTGGGAAGAGCAGCTCCCAGGAATCCCCATCGCT<br>TAGAATCAAAGCCCGTTGGCAGTCGGTGCACATCCTC<br>CCACTTCGAGCCTACGACAACCTTCTGGAAACACTTT<br>GCTATAACTATTTGCCGCTTTGCGAGCAATTGGAGCC<br>AGTGCTCAATGTCAGAGACAAGgtaaacaaaattaat<br>ctagaatagcccgagatttgaaattttcacacatgaa<br>catagtttcagGAGGACTTGGCGACATCGTTGGTTCG<br>TGTTATGTACAAACACAACCTCGCAAAGGAGTTCCTG<br>TGTGATTTGATCATGAAGGAGGTCGAGAAGCTCGACA<br>ATGATCATTTAATGTTCAGAGGAAACACACTGGCCAC<br>AAAGGCTATGGAGTCGTTTATGAAACTTGTCGCCGAC	actctcccagttattcctagtttttcaatttaattta<br>gtaactgacatgttattgaattgattgcaatgtttct<br>ccattcacctgcatttcaatcactgatggcactatta<br>atgtgagggccaatagtagaacagatcttatctacgc<br>tcatagattttaattcccaattagataagcccatcgt<br>ctcgtgtcgttctgatgactcaatctctgactagcca<br>acctttttcactttttttcgcactcaattctaaattctc<br>tctatttcttgtatcactttttttgtgtagcaatcaac<br>cattatccacgccttcagattgtc-<br>gtattgtaggtcagtagtaaattagattatcaaaatc<br>ccatacagtacaggtcgccatcaaggttacaccccga<br>cagtataagttaatccgcgttttcgattccagGTATA<br>CAGTCAACACATCTCATAGTGACTCTGGGACAAGCAG<br>AATTGCATCCGCACTAGGTGGGAAGAGCAGCTCCCAG<br>GAATCCCCATCGCTTAGAATCAAAGCCCGTTGGCAGT<br>CGGTGCACATCCTCCCACTTCGAGCCTACGACAACCT<br>TCTGGAAACACTTTGCTATAACTATTTGCCGCTTTGC<br>GAGCAATTGGAGCCAGTGCTCAATGTCAGAGACAAG

GATTATCTAGACTCAACACTCAGTGATTTTATTAAAA<br>CAGTGTTACAATGTGAGGATTCATGCGAAGTAGATCC<br>ACAGAAATTGGGTAATGTGTCAAACTCATCCCTCGAG<br>AAGAATCGTGCCCTTTTGATGCGATATGTTGAAGTGG<br>CTTGGACGAAAATTTTGAACAAgtatgttgcatgcta<br>atttcacttatgtttttatccattgaattttagCGTT<br>CACCAGCTACCAAAAAACCTCCGAGACGTATTTTCGG<br>CTCTTCGTTGCCGACTTGAAGCCCAGAATCGTGAAGC<br>TTTGGCAGATACTTTGATCTCCTCATCAATCTTCCTT<br>CGATTCTTATGTCCTGCAATTTTGAGCCCAAGCCTTT<br>TCAATCTTGTTTCCGAGTACCCATCACCAACGAACGC<br>GCGCAACTTGACTCTGATTGCCAAGACGCTGCAGAAC<br>CTTGCGAATTTCAGCAAGTTTGGAGGAAAAGAGCCTC<br>ACATGGAGTTTATGAACGAGTTTGTCGACCGAGAGTG<br>GCATAGGATGAAAGATTTCTTGTTGAGAATTTCTTCA<br>GAgtgagttgacgatgtatcacgttacataggtgcag<br>tattttgaagtactctatgtgctaaatttggcactat<br>tattaaataaatagagtataaaaatagaataacacac<br>tcacactcattgcaaagatcaaaacaattaatgacaa<br>ttttcagGTCAAAGTCGGGACCAGAAAAAAATGCGGA<br>TGCAATTGTGGACGCCGGCAAAGAGCTTAGCTTAATC<br>GCTACTTATCTTGAAGAAGCCTGGACTCCACTTCTCC<br>AAGAGAAAAATGGAAATAAGCATCCACTCTCAAATGT<br>CAAGTCGGTTTTATCCGAATTGGCAGAATGCAAGAGA<br>CGCTCGGATAATGGCGTCTTTCACTCTCCAATGGTTC<br>AACAGCCGTCTTCAGATTATGAGAATAGTCCACAGCA<br>ACACGTTGTgtgagtttcgtcttccgactttcgagtt<br>ccgagtttttgcacgcacccaccgtcattcttaacac<br>aaactaccggtcttttgtttctttaaccattatgtta<br>atacgtgtttttttcttaataaaaatttttgtctat<br>tgtagTCCTCGACATGAGAATGTACCGGCATATCGCA<br>GTACTCCGCCAACTGGCCAAGCCACGGTATTGGGCCG<br>TTCTACAAACCGCCCTGCAACTCATTTGCTCACATCG<br>GACGATTACGTCCTCTCTTCTGCATTTCAAACTCCAA<br>GTCTTCGCCCTGGAGGCACTCGGTTAAGCGATGAAAC<br>TGGCACGTCTTCGAGTCGCACCAGTGACAAGACAACC<br>AGCAGCGCTGAGATTCGAGACGACACTGATTCGGATT<br>TCGAGTTACGAGAGGATCGAGGACGTGGAGGAAGAAA<br>CCGTAAGAGGCTACCACGTACTGATGCATCACCATCG<br>AGCAGTCAACAAGCTTCAAGTGGATATTTAAGTAATA<br>ATCCTTCGAGgtaagatcacatttttctagttcaggc<br>ttctaattcggtatatgatatttcagATCCAGCTACT<br>CAAACTCTTCGAGTTCATCTCCAGTTGAACGAATGGC<br>CGCTCTATCAATTGCTAACCCAGTCTTTGGACCAGGC<br>CCATCATCTGGATATGCTATACCTGCAGAGCCAAAGG<br>AAATCGTATACCAAAAGCGAGCAAGCCCACCACCATA<br>CGATCCCGATGTGCACAACTATCATTATCAACCGgta<br>tgaattgttttcttgtattaaaaatacgttgttattt<br>ttagATGCAGGTCTACGCTGTTCCACCAGATTGTCAG<br>GTGTCCCCAAGAACGCAGGCAACAGGCGGTGTCAATG<br>CTCAGAATCGGTTAAGTCTGCCACGGACTAATCCACG<br>AGCTTCGAGGAATTCAACACTTTTGCGTCCGAGTGTC<br>GTAAATGTTCCGGATGACTGGGATAGgttatttttag<br>aggttattttaaaatctaaattaatgattttcagAAC<br>AAGTGATTACTGGAGGGACCGAGGCGAGAACAACTAC<br>CGGAGTCAACTGGAAAGCCAAGTGGAAAGTCAAGCTC<br>GAGAAATTGAACGCTTGATGAGAGAGAACATTGAGCT<br>GAAGAGTAAAATGATGTCTTCAACAAAAACTGTGGAT	

TCCAAGgtgaattttagaaaatcaaattttgatttt<br>tcaaccaaaaatagtttcagCGATCTGACAGTGGTGC<br>TAGTGAGGATTCCTACGATTCTTTGAGTTCACTCGAT<br>CGTCCATCAAGACAATCGCTTGTGGTAGTTCCAAACT<br>AAtcaatatcaaatattatcctacagttattagtctt<br>attattattttacctatttatttttatcgatctattg<br>gttcttcttgtcttaatgtaactaatgatgttcccaa<br>agaaataaaagtacatatactgtcttcccctgcactc<br>ccgttggtctcttcatttgcctgccgccaacctttcg<br>attcagtacacatcccttatctaaaggcttttcatgc<br>atattttgaatatctaaagttctcctgctcacctgtc<br>attttttgtagtttagagggtttttgattgtgatttt<br>ttaattttcaatatatgtcttcttccctccactaccc<br>atcttccgatgtacaacaacgtaaactttgtcgcact<br>gcttgtttttttagctccatacactggagatatgaaaa<br>ctacccggtagcggtgttgttagccaa	
Wild type	*gap-2(syb6873)*
acattggtcggctcatgatgtgttcccattgatatta<br>cgtagtctatgaaattatttcctcttgcgtaataatg<br>gtaatatttcgaaacatgttatttgtacttagatttc<br>aactttaaatgcaaaccattctttaaaataaaaactt<br>gtcttacgttgttatgaaattgaattattaaaaaaat<br>ctttggcccttagattaggtttttttttaattgtaatt<br>tttatcaatgtactctcccagttattcctagtttttc<br>aatttaatttagtaactgacatgttattgaattgatt<br>gcaatgtttctccattcacctgcatttcaatcactga<br>tggcactattaatgtgagggccaatagtagaacagat<br>cttatctacgctcatagattttaattcccaattagat<br>aagcccatcgtctcgtgtcgttctgatgactcaatct<br>ctgactagccaacctttttcactttttttcgcactcaat<br>tctaaattctctctatttcttgtatcactttttttgtg<br>tagcaatcaaccattatccacgccttcagattgtcAT<br>GAGAGGTGAATACGATCCATGGGATCCATCGTTTCAC<br>AATTCTTCTATGATTCCGTATTCCCTTGCTTCCATAT<br>ATCCGTCAATCGAAAATTTGCCAGAGGAATTTTCAAA<br>TGAAAATAATAAAATTAAAAATGTCTTGAAAAACTTT<br>ATTTGGTCATTCAAGCTCAAGAAAAATTACGTGCGGG<br>TATCGTCGTTGTTCGGAGAgtattgtaggtcagtagt<br>aaattagattatcaaaatcccatacagtacaggtcgc<br>catcaaggttacaccccgacagtataagttaatccgc<br>gttttcgattccagGTATACAGTCAACACATCTCATA<br>GTGACTCTGGGACAAGCAGAATTGCATCCGCACTAGG<br>TGGGAAGAGCAGCTCCCAGGAATCCCCATCGCTTAGA<br>ATCAAAGCCCGTTGGCAGTCGGTGCACATCCTCCCAC<br>TTCGAGCCTACGACAACCTTCTGGAAACACTTTGCTA<br>TAACTATTTGCCGCTTTGCGAGCAATTGGAGCCAGTG<br>CT	acattggtcggctcatgatgtgttcccattgatatta<br>cgtagtctatgaaattatttcctcttgcgtaataatg<br>gtaatatttcgaaacatgttatttgtacttagatttc<br>aactttaaatgcaaaccattctttaaaataaaaactt<br>gtcttacgttgttatgaaattgaattattaaaaaaat<br>ctttggcccttagattaggtttttttttaattgtaatt<br>tttatcaatgtactctcccagttattcctagtttttc<br>aatttaatttagtaactgacatgttattgaattgatt<br>gcaatgtttctccattcacctgcatttcaatcactga<br>tggcactattaatgtgagggccaatagtagaacagat<br>cttatctacgctcatagattttaattcccaattagat<br>aagcccatcgtctcgtgtcgttctgatgactcaatct<br>ctgactagccaacctttttcactttttttcgcactcaat<br>tctaaattctctctatttcttgtatcactttttttgtg<br>tagcaatcaaccattatccacgccttcagattgtcAT<br>GAGAGGTGAATACGACCCTTGGGACCCACTCTTTCAC<br>AATTCTTCTATGATTCCGTATTCCCTTGCTTCCATAT<br>ATCCGTCAATCGAAAATTTGCCAGAGGAATTTTCAAA<br>TGAAAATAATAAAATTAAAAATGTCTTGAAAAACTTT<br>ATTTGGTCATTCAAGCTCAAGAAAAATTACGTGCGGG<br>TATCGTCGTTGTTCGGAGAgtattgtaggtcagtagt<br>aaattagattatcaaaatcccatacagtacaggtcgc<br>catcaaggttacaccccgacagtataagttaatccgc<br>gttttcgattccagGTATACAGTCAACACATCTCATA<br>GTGACTCTGGGACAAGCAGAATTGCATCCGCACTAGG<br>TGGGAAGAGCAGCTCCCAGGAATCCCCATCGCTTAGA<br>ATCAAAGCCCGTTGGCAGTCGGTGCACATCCTCCCAC<br>TTCGAGCCTACGACAACCTTCTGGAAACACTTTGCTA<br>TAACTATTTGCCGCTTTGCGAGCAATTGGAGCCAGTG<br>CT
ZD841 background	*gap-2(syb4684)*
ataagcccatcgtctcgtgtcgttctgatgactcaat<br>ctctgactagccaacctttttcactttttttcgcactca<br>attctaaattctctctatttcttgtatcactttttttg<br>tgtagcaatcaaccattatccacgccttcagattgtc<br>ATGAGAGGTGAATACGATCCATGGGATCCATCGTTTC<br>ACAATTCTTCTATGATTCCGTATTCCCTTGCTTCCAT<br>ATATCCGTCAATCGAAAATTTGCCAGAGGAATTTTCA<br>AATGAAAATAATAAAATTAAAAATGTCTTGAAAAACT	ataagcccatcgtctcgtgtcgttctgatgactcaat<br>ctctgactagccaacctttttcactttttttcgcactca<br>attctaaattctctctatttcttgtatcactttttttg<br>tgtagcaatcaaccattatccacgccttcagattgtc<br>ATGAGAGGTGAATACGATCCATGGGATCCATCGTTTC<br>ACAATTCTTCTATGATTCCGTATTCCCTTGCTTCCAT<br>ATATCCGTCAATCGAAAATTTGCCAGAGGAATTTTCA<br>AATGAAAATAATAAAATTAAAAATGTCTTGAAAAACT

TTATTTGGTCATTCAAGCTCAAGAAAAATTACGTGCG<br>GGTAACGTCGTTGTTCGGAGAgtattgtaggtcagta<br>gtaaattagattatcaaaatcccatacagtacaggtc<br>gccatcaaggttacaccccgacagtataagttaatcc<br>gcgttttcgattccagGTATACAGTCAACACATCTCA<br>TAGTGACTCTGGGACAAGCAGAATTGCATCCGCACTA<br>GGTGGGAAGAGCAGCTCCCAGGAATCCCCATCGCTTA<br>GAATCAAAGCCCGTTGGCAGTCGGTGCACATCCTCCC<br>ACTTCGAGCCTACGACAACCTTCTGGAAACACTTTGC<br>TATAACTATTTGCCGCTTTGCGAGCAATTGGAGCCAG<br>TGCTCAATGTCAGAGACAAGgtaaacaaaattaatct<br>agaatagcccgagatttgaaattttcacacatgaaca<br>tagtttcagGAGGACTTGGCGACATCGTTGGTTCGTG<br>TTATGTACAAACACAACCTCGCAAAGGAGTTCCTGTG<br>TGATTTGATCATGAAGGAGGTCGAGAAGCTCGACAAT<br>GATCATTTAATGTTCAGAGGAAACACACTGGCCACAA<br>AGGCTATGGAGTCGT	TTATTTGGTCATTCAAGCTCAAGAAAAATTACGTGCG<br>GGTCTCGAGCTTGTTCGGCGAgtattgtaggtcagta<br>gtaaattagattatcaaaatcccatacagtacaggtc<br>gccatcaaggttacaccccgacagtataagttaatcc<br>gcgttttcgattccagGTATACAGTCAACACATCTCA<br>TAGTGACTCTGGGACAAGCAGAATTGCATCCGCACTA<br>GGTGGGAAGAGCAGCTCCCAGGAATCCCCATCGCTTA<br>GAATCAAAGCCCGTTGGCAGTCGGTGCACATCCTCCC<br>ACTTCGAGCCTACGACAACCTTCTGGAAACACTTTGC<br>TATAACTATTTGCCGCTTTGCGAGCAATTGGAGCCAG<br>TGCTCAATGTCAGAGACAAGgtaaacaaaattaatct<br>agaatagcccgagatttgaaattttcacacatgaaca<br>tagtttcagGAGGACTTGGCGACATCGTTGGTTCGTG<br>TTATGTACAAACACAACCTCGCAAAGGAGTTCCTGTG<br>TGATTTGATCATGAAGGAGGTCGAGAAGCTCGACAAT<br>GATCATTTAATGTTCAGAGGAAACACACTGGCCACAA<br>AGGCTATGGAGTCGT
	gap-2(syb4587)
	AATGCTCAGAATCGGTTAAGTCTGCCACGGACTAATC<br>CACGAGCTTCGAGGAATTCAACACTTTTGCGTCCGAG<br>TGTCGTAAATGTTCCGGATGACTGGGATAGgttattt<br>ttagaggttattttaaaatctaaattaatgattttca<br>gAACAAGTGATTACTGGAGGGACCGAGGCGAGAACAA<br>CTACCGGAGTCAACTGGAAAGCCAAGTGGAAAGTCAA<br>GCTCGAGAAATTGAACGCTTGATGAGAGAGAACATTG<br>AGCTGAAGAGTAAAATGATGTCTTCAACAAAAACTGT<br>GGATTCCAAGgtgaattttagaaaatcaaattttgat<br>tttttcaaccaaaaatagtttcagCGATCTGACAGTG<br>GTGCTAGTGAGGATTCCTACGATTCTTTGAGTTCACT<br>CGATCGTCCATCAAGACAATCGCTTGTCGTAGTTCCA<br>AACGGAGGATCAGGAGGAGGATCAGGAGGAGGATCAG<br>GAAGTAAAGGAGAAGAACTTTTCACTGGAGTTGTCCC<br>AATTCTTGTTGAATTAGATGGTGATGTTAATGGGCAC<br>AAATTTTCTGTCAGTGGAGAGGGTGAAGGTGATGCAA<br>CATACGGAAAACTTACCCTTAAATTTATTTGCACTAC<br>TGGAAAACTACCTGTTCCATGGgtaagtttaaacata<br>tatatactaactaaccctgattatttaaattttcagC<br>CAACACTTGTCACTACTTTCTgTTATGGTGTTCAATG<br>CTTcTCgAGATACCCAGATCATATGAAACgGCATGAC<br>TTTTTCAAGAGTGCCATGCCCGAAGGTTATGTACAGG<br>AAAGAACTATATTTTTCAAAGATGACGGGAACTACAA<br>GACACgtaagtttaaacagttcggtactaactaacca<br>tacatatttaaattttcagGTGCTGAAGTCAAGTTTG<br>AAGGTGATACCCTTGTTAATAGAATCGAGTTAAAAGG<br>TATTGATTTTAAAGAAGATGGAAACATTCTTGGACAC<br>AAATTGGAATACAACTATAACTCACACAATGTATACA<br>TCATGGCAGACAAACAAAAGAATGGAATCAAAGTTgt<br>aagtttaaacatgattttactaactaactaatctgat<br>ttaaattttcagAACTTCAAAATTAGACACAACATTG<br>AAGATGGAAGCGTTCAACTAGCAGACCATTATCAACA<br>AAATACTCCAATTGGCGATGGCCCTGTCCTTTTACCA<br>GACAACCATTACCTGTCCACACAATCTGCCCTTTCGA<br>AAGATCCCAACGAAAAGAGAGACCACATGGTCCTTCT<br>TGAGTTTGTAACAGCTGCTGGGATTACACATGGCATG<br>GATGAACTATACAAATAAtcaatatcaaatattatcc<br>tacagttattagtcttattattattttacctatttat<br>ttttatcgatctattggttcttcttgtcttaatgtaa

	ctaatgatgttcccaaagaaataaaagtacatatact<br>gtcttcccctgcactcccgttggtctcttcatttgcc<br>tgccgccaacctttcgattcagtacacatcccttatc<br>taaaggcttttcatgcatattttgaatatctaaagtt<br>ctcctgctcacctgtcattttttgtagtttagagggt<br>ttttgattgtgattttttaattttcaatatatgtctt<br>cttccctccactacccatcttccgatgtacaacaacg<br>taaactttgtcgcactgct
Background sequence	*gap-2(syb4587 syb5834)*
CGGATAATGTATTTTGGGGCGAGAACTTTGAGTTCAT<br>gtaagttgcaatagctcatgacatctgttaaatgggg<br>ttgctgttcaattttacatgaatagaaaatgagaaac<br>atttcaacaaccttttttttttgaaatcatcttcaac<br>acagattaactattttttggattgtcattcgaatttt<br>gaaaagatttgtgcgtcattccaaatttttaacttcg<br>attcttttttttgttttgcgaaaccaaacggaaaaag<br>tacaacagtattttccaacaccaaaaaaatttaccat<br>tcatttgtttggtatgaatgatttttccaaacgaaac<br>tttcagGATGCTCCCAAAAATCGATGAAGTGTGCGTG<br>AGCTTGTTCCGAGAATCTGATTCAAAGAAAAAGAAGG<br>ACACTCTCATCGGATACGTTACCATTGGAATCGACCA<br>ACTGTCTAGCAGAAGTCCAGTAGAGCGATGgtaagaa<br>acaagaaaaaagaaatttgtgatcattattgaaacga<br>atcacttccgatactcattcctttaggtcgttaccct<br>cgcactgaattgaatttcaatttaaagttgggaggta<br>aaactaaacaagaaaccgaatctttttctttctcctg<br>aatttaattgccttggcgacaattaactagttgatag<br>tattgcaaggagggtctattaacactccttccctcct<br>cttcaactgaaaacgattctctcgaaacgaaaatgcg<br>atttggtttcggttttgtgtctctatgccagattgtc<br>ggcttgttagtctgttagtcataggcaaccccccatt<br>ggtcgtaacagatagatatgcagtaggtctataactt<br>cgcattaacctccttgttcggatcaaaagtcgctct	CGGATAATGTATTTTGGGGCGAGAACTTTGAGTTCAT<br>gtaagttgcaatagctcatgacatctgttaaatgggg<br>ttgctgttcaattttacatgaatagaaaatgagaaac<br>atttcaacaaccttttttttttgaaatcatcttcaac<br>acagattaactattttttggattgtcattcgaatttt<br>gaaaagatttgtgcgtcattccaaatttttaacttcg<br>attcttttttttgttttgcgaaaccaaacggaaaaag<br>tacaacagtattttccaacaccaaaaaaatttaccat<br>tcatttgtttggtatgaatgatttttccaaacgaaac<br>tttcagGATGCTCCCAAAAATCGATGAAGTGTGCGTG<br>AGCTTGTTCCGAGAATCTGATTCAAAGAAAAAGAAGG<br>ACACTCTCATCTAGAACGTTACCATTGGAATCGACCA<br>ACTGTCTAGCAGAAGTCCAGTAGAGCGATGgtaagaa<br>acaagaaaaaagaaatttgtgatcattattgaaacga<br>atcacttccgatactcattcctttaggtcgttaccct<br>cgcactgaattgaatttcaatttaaagttgggaggta<br>aaactaaacaagaaaccgaatctttttctttctcctg<br>aatttaattgccttggcgacaattaactagttgatag<br>tattgcaaggagggtctattaacactccttccctcct<br>cttcaactgaaaacgattctctcgaaacgaaaatgcg<br>atttggtttcggttttgtgtctctatgccagattgtc<br>ggcttgttagtctgttagtcataggcaaccccccatt<br>ggtcgtaacagatagatatgcagtaggtctataactt<br>cgcattaacctccttgttcggatcaaaagtcgctct

Table S3. Sequence information of strains generated by CRISPR-Cas9 genome editing (40)

REFERENCES AND NOTES

1. H. E. Hoekstra, G. E. Robinson, Behavioral genetics and genomics: Mendel's peas, mice, and bees. *Proc. Natl. Acad. Sci. U.S.A.* **119**, e2122154119 (2022).

2. N. Niepoth, A. Bendesky, How natural genetic variation shapes behavior. *Annu. Rev. Genomics Hum. Genet.* **21**, 437–463 (2020).

3. C. M. Bubac, J. M. Miller, D. W. Coltman, The genetic basis of animal behavioural diversity in natural populations. *Mol. Ecol.* **29**, 1957–1971 (2020).

4. E. L. Yap, M. E. Greenberg, Activity-regulated transcription: Bridging the gap between neural activity and behavior. *Neuron* **100**, 330–348 (2018).

5. M. S. Hill, P. Vande Zande, P. J. Wittkopp, Molecular and evolutionary processes generating variation in gene expression. *Nat. Rev. Genet.* **22**, 203–215 (2021).

6. GTEx Consortium, Genetic effects on gene expression across human tissues. *Nature* **550**, 204–213 (2017).

7. N. de Klein, E. A. Tsai, M. Vochteloo, D. Baird, Y. Huang, C. Y. Chen, S. van Dam, R. Oelen, P. Deelen, O. B. Bakker, O. el Garwany, Z. Ouyang, E. E. Marshall, M. I. Zavodszky, W. van Rheenen, M. K. Bakker, J. Veldink, T. R. Gaunt, H. Runz, L. Franke, H. J. Westra, Brain expression quantitative trait locus and network analyses reveal downstream effects and putative drivers for brain-related diseases. *Nat. Genet.* **55**, 377–388 (2023).

8. G. Zhang, N. M. Roberto, D. Lee, S. R. Hahnel, E. C. Andersen, The impact of species-wide gene expression variation on *Caenorhabditis elegans* complex traits. *Nat. Commun.* **13**, 3462 (2022).

9. W. S. Schackwitz, T. Inoue, J. H. Thomas, Chemosensory neurons function in parallel to mediate a pheromone response in *C. elegans*. *Neuron* **17**, 719–728 (1996).

10. P. Ren, C. S. Lim, R. Johnsen, P. S. Albert, D. Pilgrim, D. L. Riddle, Control of *C. elegans* larval development by neuronal expression of a TGF-β homolog. *Science* **274**, 1389–1391 (1996).

11. J. Ben Arous, S. Laffont, D. Chatenay, Molecular and sensory basis of a food related two-state behavior in *C. elegans*. *PLOS ONE* **4**, e7584 (2009).

12. E. R. Greer, C. L. Pérez, M. R. van Gilst, B. H. Lee, K. Ashrafi, Neural and molecular dissection of a *C. elegans* sensory circuit that regulates fat and feeding. *Cell Metab.* **8**, 118–131 (2008).

13. S. Stern, C. Kirst, C. I. Bargmann, Neuromodulatory control of long-term behavioral patterns and individuality across development. *Cell* **171**, 1649–1662 (2017).

14. K. Milward, K. E. Busch, R. J. Murphy, M. de Bono, B. Olofsson, Neuronal and molecular substrates for optimal foraging in *Caenorhabditis elegans*. *Proc. Natl. Acad. Sci. U.S.A.* **108**, 20672–20677 (2011).

15. Y. J. You, J. Kim, D. M. Raizen, L. Avery, Insulin, cGMP, and TGF-β signals regulate food intake and quiescence in *C. elegans*: A model for satiety. *Cell Metab.* **7**, 249–257 (2008).

16. S. A. Boor, J. D. Meisel, D. H. Kim, Neuroendocrine gene expression coupling of interoceptive bacterial food cues to foraging behavior of *C. elegans*. *eLife* **12**, RP91120 (2024).

17. M. Fletcher, D. H. Kim, Age-dependent neuroendocrine signaling from sensory neurons modulates the effect of dietary restriction on longevity of *Caenorhabditis elegans*. *PLOS Genet.* **13**, e1006544 (2017).

18. J. D. Meisel, O. Panda, P. Mahanti, F. C. Schroeder, D. H. Kim, Chemosensation of bacterial secondary metabolites modulates neuroendocrine signaling and behavior of *C. elegans*. *Cell* **159**, 267–280 (2014).

19. E. V. Entchev, D. S. Patel, M. Zhan, A. J. Steele, H. Lu, Q. L. Chng, A gene-expression-based neural code for food abundance that modulates lifespan. *eLife* **4**, e06259 (2015).

20. M. P. O'Donnell, P. H. Chao, J. E. Kammenga, P. Sengupta, Rictor/TORC2 mediates gut-to-brain signaling in the regulation of phenotypic plasticity in *C. elegans*. *PLOS Genet.* **14**, e1007213 (2018).

21. Z. A. Hilbert, D. H. Kim, Sexually dimorphic control of gene expression in sensory neurons regulates decision-making behavior in *C. elegans*. *eLife* **6**, e21166 (2017).

22. D. E. Cook, S. Zdraljevic, J. P. Roberts, E. C. Andersen, CeNDR, the *Caenorhabditis elegans* natural diversity resource. *Nucleic Acids Res.* **45**, D650–D657 (2017).

23. M. Fujiwara, P. Sengupta, S. L. McIntire, Regulation of body size and behavioral state of *C. elegans* by sensory perception and the egl-4 cGMP-dependent protein kinase. *Neuron* **36**, 1091–1102 (2002).

24. A. Bendesky, J. Pitts, M. V. Rockman, W. C. Chen, M. W. Tan, L. Kruglyak, C. I. Bargmann, Long-range regulatory polymorphisms affecting a GABA receptor constitute a quantitative trait locus (QTL) for social behavior in *Caenorhabditis elegans*. *PLOS Genet.* **8**, e1003157 (2012).

25. M. De Bono, C. I. Bargmann, Natural variation in a neuropeptide Y receptor homolog modifies social behavior and food response in *C. elegans*. *Cell* **94**, 679–689 (1998).

26. S. Hayashizaki, Y. Iino, M. Yamamoto, Characterization of the *C. elegans* gap-2gene encoding a novel Ras-GTPase activating protein and its possible role in larval development. *Genes Cells* **3**, 189–202 (1998).

27. C. I. Bargmann, Beyond the connectome: How neuromodulators shape neural circuits. *Bioessays* **34**, 458–465 (2012).

28. S. W. Flavell, N. Pokala, E. Z. Macosko, D. R. Albrecht, J. Larch, C. I. Bargmann, Serotonin and the neuropeptide PDF initiate and extend opposing behavioral states in *C. elegans*. *Cell* **154**, 1023–1035 (2013).

29. M. D. Gyurkó, P. Csermely, C. Soti, A. Steták, Distinct roles of the RasGAP family proteins in *C. elegans* associative learning and memory. *Sci. Rep.* **5**, 15084 (2015).

30. A. Klose, M. R. Ahmadian, M. Schuelke, K. Scheffzek, S. Hoffmeyer, A. Gewies, F. Schmitz, D. Kaufmann, H. Peters, A. Wittinghofer, P. Nürnberg, Selective disactivation of neurofibromin GAP activity in neurofibromatosis type 1. *Hum. Mol. Genet.* **7**, 1261–1268 (1998).

31. S. Gretarsdottir, A. F. Baas, G. Thorleifsson, H. Holm, M. den Heijer, J.P. P. M. de Vries, S. E. Kranendonk, C. J. A. M. Zeebregts, S. M. van Sterkenburg, R. H. Geelkerken, A. M. van Rij, M. J. A. Williams, A. P. M. Boll, J. P. Kostic, A. Jonasdottir, A. Jonasdottir, G. B. Walters, G. Masson, P. Sulem, J. Saemundsdottir, M. Mouy, K. P. Magnusson, G. Tromp, J. R. Elmore, N. Sakalihasan, R. Limet, J. O. Defraigne, R. E. Ferrell, A. Ronkainen, Y. M. Ruigrok, C. Wijmenga, D. E. Grobbee, S. H. Shah, C. B. Granger, A. A. Quyyumi, V. Vaccarino, R. S. Patel, A. M. Zafari, A. I. Levey, H. Austin, D. Girelli, P. F. Pignatti, O. Olivieri, N. Martinelli, G. Malerba, E. Trabetti, L. C. Becker, D. M. Becker, M. P. Reilly, D. J. Rader, T. Mueller, B. Dieplinger, M. Haltmayer, S. Urbonavicius, B. Lindblad, A. Gottsäter, E. Gaetani, R. Pola, P. Wells, M. Rodger, M. Forgie, N. Langlois, J. Corral, V. Vicente, J. Fontcuberta, F. España, N. Grarup, T. Jørgensen, D. R. Witte, T. Hansen, O. Pedersen, K. K. Aben, J. de Graaf, S. Holewijn, L. Folkersen, A. Franco-Cereceda, P. Eriksson, D. A. Collier, H. Stefansson, V. Steinthorsdottir, T. Rafnar, E. M. Valdimarsson, H. B. Magnadottir, S. Sveinbjornsdottir, I. Olafsson, M. K. Magnusson, R. Palmason, V. Haraldsdottir, K. Andersen, P. T. Onundarson, G. Thorgeirsson, L. A. Kiemeney, J. T. Powell, D. J. Carey, H. Kuivaniemi, J. S. Lindholt, G. T. Jones, A. Kong, J. D. Blankensteijn, S. E. Matthiasson, U. Thorsteinsdottir, K. Stefansson, Genome-wide association study identifies a sequence variant within the DAB2IP gene conferring susceptibility to abdominal aortic aneurysm. *Nat. Genet.* **42**, 692–697 (2010).

32. M. Hamakawa, T. Uozumi, N. Ueda, Y. Iino, T. Hirotsu, A role for Ras in inhibiting circular foraging behavior as revealed by a new method for time and cell-specific RNAi. *BMC Biol.* **13**, 6 (2015).

33. T. Janssen, S. J. Husson, E. Meelkop, L. Temmerman, M. Lindemans, K. Verstraelen, S. Rademakers, I. Mertens, M. Nitabach, G. Jansen, L. Schoofs, Discovery and characterization of a conserved pigment dispersing factor-like neuropeptide pathway in *Caenorhabditis elegans*. *J. Neurochem.* **111**, 228–241 (2009).

34. J. Sulston, M. Dew, S. Brenner, Dopaminergic neurons in the nematode *Caenorhabditis elegans*. *J Comp Neurol* **163**, 215–226 (1975).

35. M. A. Félix, C. Braendle, The natural history of *Caenorhabditis elegans*. *Curr. Biol.* **20**, R965–R969 (2010).

36. D. H. Kim, S. W. Flavell, Host-microbe interactions and the behavior of *Caenorhabditis elegans*. *J. Neurogenet.* **34**, 500–509 (2020).

37. S. C. Brady, S. Zdraljevic, K. W. Bisaga, R. E. Tanny, D. E. Cook, D. Lee, Y. Wang, E. C. Andersen, A novel gene underlies bleomycin-response variation in *Caenorhabditis elegans*. *Genetics* **212**, 1453–1468 (2019).

38. N. E. Morton, Logarithm of odds (lods) for linkage in complex inheritance. *Proc. Natl. Acad. Sci. U.S.A.* **93**, 3471–3476 (1996).

39. J. Schindelin, I. Arganda-Carreras, E. Frise, V. Kaynig, M. Longair, T. Pietzsch, S. Preibisch, C. Rueden, S. Saalfeld, B. Schmid, J. Y. Tinevez, D. J. White, V. Hartenstein, K. Eliceiri, P. Tomancak, A. Cardona, Fiji: An open-source platform for biological-image analysis. *Nat. Methods* **9**, 676–682 (2012).

40. D. J. Dickinson, A. M. Pani, J. K. Heppert, C. D. Higgins, B. Goldstein, Streamlined genome engineering with a self-excising drug selection cassette. *Genetics* **200**, 1035–1049 (2015).
